# Intratumor *Lactobacillus* drives ferroptosis resistance via D-lactate-STAT3 K631 lactylation in esophageal squamous cell carcinoma

**DOI:** 10.1080/19490976.2026.2685912

**Published:** 2026-06-11

**Authors:** Dong Wang, Heng Lu, Weiguang Li, Liuying Li, Xuesen Xu, Huimin Zhou, Hui Tao, Yizhen Li, Wei Wei, Kunpeng Wu, Yihan Wang, Run Li, Yuzhong Li, Weiyan Yao, Yanwen Chen, Fangyu Wang, Lei Ye

**Affiliations:** a Department of Gastroenterology, Ruijin Hospital, Shanghai Jiao Tong University School of Medicine, Shanghai, China; b State Key Laboratory of Natural Medicines, School of Pharmacy, China Pharmaceutical University, Nanjing, China; c Department of Gastroenterology and Hepatology, Jinling Hospital, Affiliated Hospital of Medical School, Nanjing University, Nanjing, China; d Department of Esophageal Surgery, Department of Thoracic Surgery, Nanjing Drum Tower Hospital, The Affiliated Hospital of Nanjing University Medical School, Nanjing, China; e Department of Thoracic Surgery, Nanjing Lishui People's Hospital, Zhongda Hospital Lishui Branch, Southeast University, Nanjing, China

**Keywords:** Intratumor bacteria, esophageal squamous cell carcinoma, *Lactobacillus*, lactylation, ferroptosis

## Abstract

Tumor-resident microbiota are increasingly recognized as active components of the gastrointestinal tumor ecosystem, yet how intratumor bacteria reshape cancer cell stress responses in esophageal squamous cell carcinoma (ESCC) remains unclear. Here, 16S rRNA gene sequencing of 102 multiregional tissue blocks from 27 patients with ESCC, integrated with untargeted metabolomics, RNA sequencing, and mass spectrometry, identified *Lactobacillus*, particularly *Lactobacillus reuteri*, as a tumor-enriched taxon associated with adverse survival. Mechanistic studies in ESCC cell lines and nude-mouse xenografts revealed a distinctive dynamic: in contrast to host-derived L-lactate, *L. reuteri*-derived D-lactate induced site-specific STAT3 lactylation at lysine 631, thereby promoting STAT3 dimerization and nuclear translocation. This host signaling rewiring upregulated the ferroptosis suppressors GPX4 and FTH1, reduced ferroptotic vulnerability, and enhanced ESCC growth. Disrupting bacterial D-lactate production using a *ldhD*-deficient *L. reuteri* mutant or blocking host STAT3 lactylation using STAT3-knockout cells reconstituted with wild-type STAT3 or lactylation-defective STAT3 K631R, abolished the pro-tumor and antiferroptotic effects *in vitro* and *in vivo*. Together, these findings define a tumor-resident microbe–metabolite–host signaling axis that links intratumor *Lactobacillus* to ferroptosis escape. By establishing bacteria-derived D-lactate as the functional driver, the study provides a novel mechanistic framework that extends beyond the classical Warburg effect for developing biomarkers and therapeutic strategies targeting STAT3 lactylation or ferroptosis sensitization. These translatable results emphasize the need for context-specific evaluation of *Lactobacillus*-containing probiotic supplementation in patients with ESCC.

## Introduction

Esophageal cancer ranks sixth in cancer-related mortality and seventh in incidence worldwide, with esophageal squamous cell carcinoma (ESCC) representing the predominant histological subtype in Eastern Asia.^1-3^ While historical research has predominantly focused on host genetic alterations, accumulating evidence indicates that the tumor mass is not an isolated entity of malignant cells but a complex ecosystem heavily influenced by its local microenvironment. This shifting paradigm underscores the need to define tumor-intrinsic and microenvironmental determinants of ESCC progression and therapy response.

Accumulating evidence indicates that solid tumors, including ESCC, harbor distinct and viable intratumor microbiota that profoundly influence tumor biology and clinical outcomes.^4-6^ While prior studies in ESCC have linked specific taxa (e.g., *Fusobacterium nucleatum* and *Porphyromonas gingivalis*) to poor prognosis and immune evasion,^7-9^ the biological functions of intratumor *Lactobacillus* remain poorly understood. *Lactobacillus* species are widely regarded as beneficial probiotics in gut health, yet their effects are context-dependent. Recent clinical evidence reveals that high intratumor *Lactobacillus* abundance is characteristic of high-grade esophageal dysplasia^10^ and correlates with poor long-term survival in patients with ESCC.^11^ This seemingly abrupt shift from a “beneficial commensal” to a potential “pro-tumoral factor” reflects niche-specific biology. The presence of *Lactobacillus* in the esophagus stems from multiple routes: descending oral transmission, ascending gastric acid reflux (predominating in the lower esophagus),^3^ and potentially vascular dissemination via disrupted epithelial barriers.^4^ Once colonized, the hypoxic and acidic tumor microenvironment selectively favors these acid-tolerant lactic acid bacteria, reshaping local host-microbe metabolic crosstalk to promote tumorigenesis.

A central element of this host-microbe crosstalk is metabolic reprogramming. While host-derived L-lactate is recognized as a mediator of the traditional Warburg effect, the functional significance of microbiota-derived lactate, particularly D-lactate, which is comparatively low in mammalian cells, remains elusive.^12^ Metabolites fundamentally reprogram tumor phenotypes through posttranslational modifications (PTMs). Signal transducer and activator of transcription 3 (STAT3), a classical oncogene frequently activated in ESCC, is heavily modulated by PTMs.^13^ ^,^ ^14^ Yet, whether intratumor bacteria utilize D-lactate to regulate STAT3 activity and alter ESCC stress responses is unknown.

A highly relevant cellular stress response in this context is ferroptosis, an iron-dependent form of regulated cell death characterized by dysregulated glutathione biosynthesis and lipid peroxidation.^15^ ESCC exhibits a unique biological reliance on ferroptosis-escape mechanisms compared to other gastrointestinal malignancies. ESCC etiology is strongly driven by potent exogenous reactive oxygen species (ROS) insults (e.g., intensive smoking and alcohol consumption) and is characterized by distinct lipid metabolism reprogramming.^16^ To survive this intrinsically hostile, high-oxidative-stress environment, ESCC tumors must establish exceedingly robust antioxidative and antiferroptotic defense networks.^17^ Consequently, preclinical ESCC models position ferroptosis as a compelling therapeutic target for ESCC therapy.^18^

Here, by profiling 102 multiregional tissue blocks from 27 patients with ESCC, we reveal substantial spatial heterogeneity of the intratumor microbiota and identify *Lactobacillus*, particularly *L. reuteri*, as a tumor-enriched taxon associated with adverse clinical outcome. Integrating multi-omics with genetic perturbation, we show that *L. reuteri*–derived D-lactate, induces site-specific STAT3 K631 lactylation, thereby promoting STAT3 dimerization and nuclear translocation as well as upregulating the ferroptosis suppressors GPX4 and FTH1. This microbiota–metabolite–PTM axis suppresses ferroptosis and enhances ESCC tumor growth both *in vitro* and *in vivo*. By distinguishing microbiota-derived D-lactate from host-derived L-lactate and positioning D-lactate as a functional driver, our study extends beyond classical Warburg-focused paradigms. In summary, our study establishes a testable clinical framework, prompting a critical re-evaluation of *Lactobacillus*-based probiotic use in ESCC patients and highlighting metabolic or PTM-targeted therapeutic avenues to restore ferroptosis sensitivity.

## Methods

Detailed protocols for bacterial culture, FISH, TEM, and other *in vitro* and *in vivo* assays can be found in the online Supplemental materials.

### Key reagents and resources

The principal reagents, cell lines, antibodies, and deposited data sets utilized in the study are listed in Supplementary Table S1.

### Patient cohort and clinical specimens

A total of 27 patients with histologically confirmed ESCC who underwent surgical resection at Jinling Hospital (Affiliated Hospital of Medical School, Nanjing University, P. R. China) were enrolled. Patients receiving prior neoadjuvant chemo- or radiotherapy were excluded to avoid therapy-induced impact on tissue-resident microbiota. Clinical characteristics including age, sex, smoking, and alcohol history, pathological TNM stage, are provided in Supplementary Table S2. Overall survival (OS) and progression-free survival (PFS) were defined from the date of surgery to the date of death or progression or last follow-up (cutoff date: December 31, 2024).

Fresh ESCC tissues and morphologically normal adjacent tissues (NATs) were obtained immediately postresection. Tissue sampling strictly adhered to a predefined sterile workflow. Each tumor block was dissected into multidimensional spatial fragments under sterile conditions to capture intratumoral heterogeneity. Tissues were immediately flash-frozen in sterile 5-mL conical tubes with sterile RPMI-1640 medium (Gibco, USA) using liquid nitrogen, and subsequently stored at −80 °C. This study protocol was approved by the Ethical Committee of Jinling Hospital (Approval No: 2023DZKY-022-01), and written informed consent was obtained from all participants.

### Cell lines

Human ESCC cell lines (Eca-109 and TE-1) were maintained in RPMI-1640 medium (Gibco) supplemented with standard additives. Cell line identity was authenticated by STR profiling prior to experimentation. Neither line has been previously reported as misidentified or contaminated, and regular testing confirmed they were mycoplasma-free.

### Low-biomass tumor microbiome profiling and rigorous contamination controls

We implemented rigorous contamination controls throughout sampling, DNA extraction, library preparation, and bioinformatic processing.

*DNA extraction and Amplification*: Genomic DNA was extracted from frozen tissues (40–70 mg) using the CTAB method^4^ in a dedicated, ultra-clean biosafety cabinet, ensuring complete physical separation from post-PCR environments. Crucially, nontemplate controls (NTC), DNA extraction blanks (reagents only), and environmental swabs from the dissection area were processed in parallel across every batch.

*16S rRNA Sequencing:* The 16S rRNA gene amplification was performed by targeting five hypervariable regions (V2, V3, V5, V6, and V8). Libraries were sequenced on an Illumina NovaSeq 6000 system (PE150 mode).

*Computational Decontamination*: Raw reads were quality-filtered using fqtrim (v0.94), discarding reads <100 bp or those with an average window quality score < 20. Clean reads were merged and taxonomically profiled using the short multiple regions framework (SMURF) against a refined Greengenes database (May 2013 version, with in-house refinements). Taxa exhibiting a prevalence higher than 30% in negative controls were defined as experimental artifacts and rigorously filtered from all biological samples using standard tumor microbiome (TMB) decontamination pipelines.^4^ The detailed list of detected contaminants is reported in Supplementary Table S3. Ultimately, the authentic presence of tumor-resident *Lactobacillus* was confirmed orthogonally via FISH, TEM, and live bacterial culture.

### Microbiome data analysis

Alpha diversity was evaluated utilizing Shannon indices, while beta diversity was computed based on the Bray–Curtis distance matrix and visualized via principal coordinate analysis (PCoA). Differential abundance analysis was conducted using Fisher's exact test for samples without biological replicates, Mann–Whitney *U* test for two-group comparisons with biological replicates, and Kruskal–Wallis test for multigroup comparisons with biological replicates, with significance defined as *P* < 0.05. Bioinformatics pipelines were executed utilizing OmicStudio tools (http://www.omicstudio.cn/tool).

### Statistical analysis

Unless otherwise stated, experiments were independently repeated at least three times. Data were analyzed using R (version 4.5.1) and GraphPad Prism 9, and are presented as means ± SEM. Differences between two independent groups were analyzed using an unpaired two-tailed Student's t-test, with Welch's correction applied for unequal variances. Nonnormally distributed variables (e.g., relative microbial abundance) were evaluated using the Mann‒Whitney U test. Paired clinical specimens were analyzed via the paired two-tailed Student's t-test. For multiple comparisons, a one-way analysis of variance (ANOVA) followed by Tukey's post hoc test was performed. Predefined pairwise comparisons across multiple conditions were conducted using independent Student's t-tests. Time-course experiments were evaluated at the terminal endpoint using an unpaired Student's *t*-test. Correlations between variables were determined using Spearman's rank correlation. Survival analyzes were conducted at the patient level.

For patient-level survival analyzes, in cases of multiregional spatial sampling, the *Lactobacillus* abundance for a given patient was aggregated using the within-patient mean. To avoid outcome-dependent cutoff optimization bias, patients were stratified into high and low intratumor *Lactobacillus* abundance groups according to the median relative abundance across the entire cohort. Survival distributions were compared employing the log-rank test. Associations with OS and PFS were assessed using multivariable Cox proportional-hazards models, and median-dichotomized *Lactobacillus* abundance, age, sex, and pathological TNM stage were included in the adjusted models, with the low-*Lactobacillus* abundance group used as the reference. To validate the prognostic robustness of *Lactobacillus* abundance and mitigate potential intratumor spatial sampling bias, a computational bootstrap resampling approach was employed. Clinical datasets from GDC, TIMER, and GEPIA2 were queried to benchmark GPX4 and FTH1 transcriptomic profiles in ESCC. A probability threshold of *P* < 0.05 was defined as statistically significant.

## Results

### Tumor-resident *Lactobacillus* is enriched in ESCC and associates with poor outcomes

**Figure 1.**
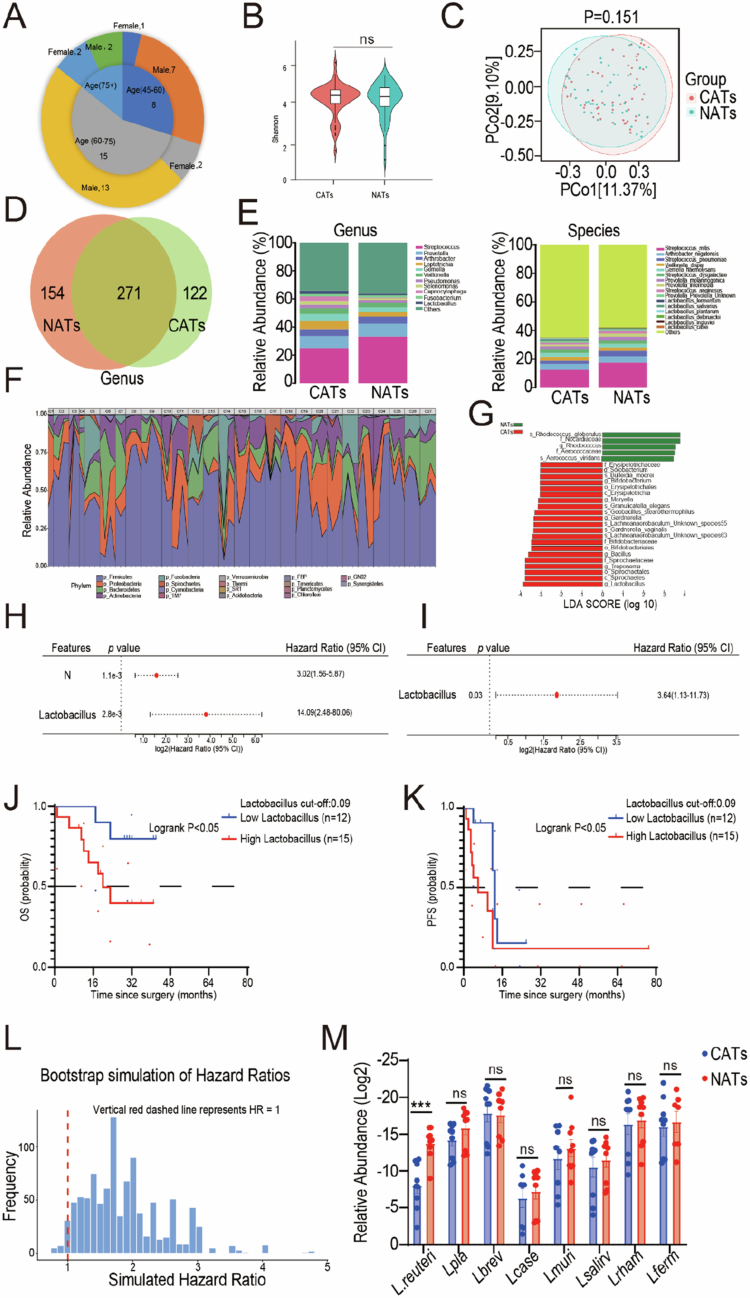
Tumor-resident *Lactobacillus* is enriched in ESCC and associates with poor outcomes. (A) Number of included patients and distribution of biological sex by age. (B) Shannon index of CATs and NATs. Wilcoxon rank-sum test (*P* > 0.05). (C) Unsupervised PCoA plot using Bray–Curtis distance for all samples. ANOSIM test (*P* > 0.05). (D) Venn diagram of genera in CATs and NATs. (E) Stacked bar diagrams of the relative abundance of bacterial communities in CATs and NATs at the genus and species levels. (F) Bacterial distribution of the top 18 phyla across patients. The microbiome constitutions of CATs and NATs were combined for each patient; patients are abbreviated as “C”. (G) Bar plot of linear discriminant analysis effect size (LEfSe) scores for taxa differentially enriched in CATs versus NATs (LDA > 3.0). (H, I) Multivariate Cox proportional-hazards regression analysis for overall survival and progression-free survival in ESCC. Forest plots displaying the Hazard Ratios (HR) and 95% Confidence Intervals (CI) for variables including lymph node metastasis status and median-dichotomized *Lactobacillus* abundance. An HR > 1 indicates a higher risk of mortality or progression (*P* < 0.05). (J, K) Kaplan–Meier overall survival(J) and progression-free survival (K) curves of patients with ESCC stratified by low versus high intratumor *Lactobacillus* abundance (median cutoff = 0.09). Log-rank test (*P* < 0.05). (L) Histogram illustrating the density distribution of simulated hazard ratios calculated exclusively from 994 convergent iterations of a computational bootstrap resampling model mimicking single-lesion biopsy events. The vertical red dashed line marks the threshold of entirely no effect (HR = 1). (M) qPCR quantification comparing the abundance of *L. reuteri* in paired CATs and NATs. Note that the Y-axis portrays negative Log2 values; higher values (closer to 0) indicate higher relative bacterial abundance. Paired two-tailed Student's t-test (*n* = 7–9). Data are presented as means ± SEM. “*n*” represents the number of biologically independent tissue samples or patients in each group. ***P < 0.001; ns, not significant. Abbreviations: CATs, cancer tissues; NATs, normal adjacent tissues; PCoA, principal coordinate analysis; ANOSIM, analysis of similarities; qPCR, quantitative PCR; LDA, linear discriminant analysis; HR, Hazard ratio.

Twenty-seven ESCC patients contributed 102 multiregional tissue samples, comprising approximately two CATs and two matched NATs per patient (Figure 1A and Supplementary Table S2). We profiled the intratumor microbiota by 16S rRNA gene sequencing. Alpha and beta diversity between CATs and NATs were similar (Figure 1B-C). At the genus level, 271 genera were shared between CATs and NATs (Figure 1D). Despite comparable diversity, overall community composition differed between tissue types: CATs exhibited a reduced relative abundance of *Streptococcus* and a comprehensively increased relative abundance of *Lactobacillus* (Figure 1E).

At the phylum level, the tumor-resident microbiota was principally dominated by *Firmicutes* (mean 50.57%), followed by *Proteobacteria* (mean 17.06%), and *Bacteroidetes* (mean 11.94%) (Figure 1F). Linear discriminant analysis effect size (LEfSe) identified *Lactobacillus* as the principal discriminatory taxon specifically enriched in CATs (Figure 1G, Supplementary Figure S1A-B).

Microbial composition varied spatially across esophageal regions (Supplementary Figure S1C). *Lactobacillus* was predominantly located in the upper and lower esophagus, with relatively lower abundance in the middle esophagus. Redundancy analysis suggested positive associations between *Lactobacillus* abundance and serum carcinoembryonic antigen levels and dietary sugar intake (Supplementary Figure S1D). Crucially, as shown in Figure 1H-I, an exploratory stepwise multivariable Cox regression including median-dichotomized *Lactobacillus* abundance, age, sex and pathological TNM stage as candidate variables, high *Lactobacillus* abundance was retained in the final model and was associated with increased risk of death and disease progression/recurrence, respectively (OS HR:14.09, 95% CI 2.48–80.06, *P* < 0.01; PFS HR:3.64, 95% CI 1.13–11.73, *P* < 0.05, Supplementary Table S4). Kaplan‒Meier curves using a prespecified median split yielded concordant results (log-rank *P* < 0.05, Figure 1J-K). Recognizing that multiregional sampling within a relatively limited patient cohort (*n* = 27) may introduce spatial clustering bias, we sought to verify whether this prognostic trend holds true if only a single, random clinical biopsy were taken per patient. We performed a rigorous 1000-iteration bootstrap resampling analysis to computationally simulate single random sampling events. Out of 994 successfully converged iteration models, an overwhelming 96.2% demonstrated a consistent adverse survival trend (HR > 1) for the high *Lactobacillus* group, yielding a median simulated hazard ratio of 1.743 (Figure 1L). This computational validation supports the directional stability of the adverse prognostic association and suggests that it is unlikely to be solely driven by spatial sampling bias. Species-level taxonomic profiling suggested a specific enrichment of *L. reuteri* within tumor tissues (Supplementary Figure S1E). To validate this, we then designed primers for eight common *Lactobacillus* species and quantified their abundance in paired tissues via qPCR, and only *L. reuteri* was significantly enriched in CATs (Figure 1M).

Consistent with prior reports on intratumor microbial heterogeneity,^19^ most patients contributed two or three spatially separated tissue blocks with matched NATs (collected ≥ 2 cm from the tumor margin and confirmed tumor-free by pathology). Individual tumor blocks exhibited distinct microbial profile, differing both from paired NATs and from other blocks within the same tumor microenvironment (Supplementary Figure S2). Collectively, these data substantiate that *Lactobacillus*, particularly *L. reuteri,* is a clinically relevant and negatively prognostic component of the ESCC tumor microbiome.

### Intratumor *Lactobacillus* abundance positively correlates with elevated microenvironmental lactate

**Figure 2.**
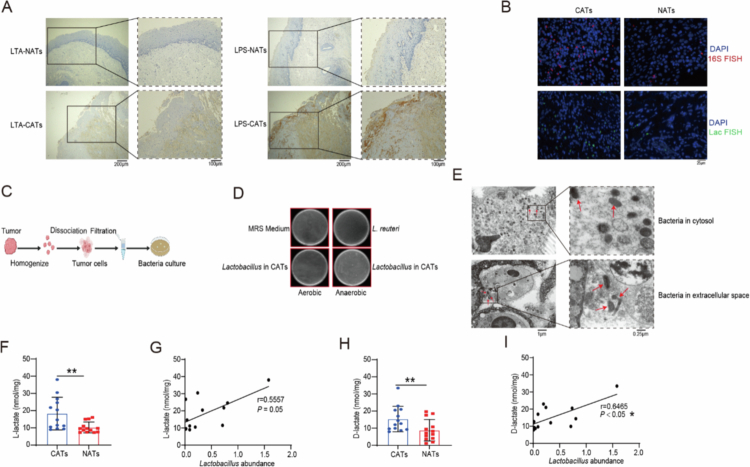
Orthogonal validation of living tumor-resident *Lactobacillus* and its correlation with intratumor lactate. (A) Immunohistochemistry (IHC) staining of CATs and NATs with anti-LPS or anti-LTA antibodies to assess Gram-negative bacteria or Gram-positive bacteria, respectively. Scale bars from left to right, 200 and 100 μm. (B) Fluorescence in situ hybridization (FISH) of CATs and NATs showing *Lactobacillus* distribution; a universal 16S rRNA probe was used as a positive control. Red, 16S probe; green, *Lactobacillus* probe; blue, DAPI. Scale bar, 25 μm. (C) Scheme outlining bacterial culture isolation from CAT samples. (D) Representative culture plate images of *Lactobacillus* isolated from CATs under both aerobic and anaerobic conditions. A standard *L. reuteri* strain was utilized as the positive biological control. (E) TEM images showing bacteria localized in both the cytosol (top panels) and the extracellular space (bottom panels) of CATs. Red arrows denote bacterial structures. Scale bars from left to right, 1 and 0.25 μm. (F, H) Concentrations of L-lactate (F) and D-lactate (H) in CATs versus NATs measured using commercial kits (*n* = 13). Paired two-tailed Student's t-test. (G, I) Spearman's rank correlation between concentrations of L-lactate (G) or D-lactate (I) and intratumor *Lactobacillus* abundance (*n* = 13; correlation statistics shown in the plots). Data are presented as means ± SEM. “*n*” represents the number of biologically independent tissue samples in each group. *P < 0.05, **P < 0.01. Abbreviations: CATs, cancer tissues; NATs, normal adjacent tissues; LPS, lipopolysaccharide; LTA, lipoteichoic acid; FISH, fluorescence *in situ* hybridization; TEM, transmission electron microscope.

To robustly validate the physical presence of live bacteria within tumors and exclude abiotic DNA contamination, we conducted orthogonal biological validations. Immunohistochemistry (IHC) using universal lipopolysaccharide (LPS, targeting Gram-negative) and lipoteichoic acid (LTA, targeting Gram-positive) antibodies demonstrated a pervasive bacterial infiltration in ESCC specimens, with visibly denser bacterial burdens in CATs compared to matched NATs (Figure 2A). We next conducted RNA fluorescence *in situ* hybridization (FISH) using a universal bacterial 16S rRNA probe or a *Lactobacillus*-specific probe to localize bacteria in CATs and NATs. Bacterial signals were detected in close proximity to esophageal epithelial cells, and *Lactobacillus* signals were significantly higher in CATs than in NATs (Figure 2B). To ascertain bacterial viability, we homogenized CATs under strict aseptic manipulation and processed the tissue suspensions for live bacterial isolation (Figure 2C). Cultivation on *Lactobacillus*-selective MRS agar demonstrated that tumor-derived *Lactobacillus* remained strictly viable and replicative exclusively under anaerobic microenvironments, phenocopying the standard *L. reuteri* control strain (Figure 2D). Furthermore, high-resolution transmission electron microscopy (TEM) identified morphologically intact bacteria-like structures localized both intracellularly and in the extracellular matrix (Figure 2E).

Given that *Lactobacillus* can produce both L- and D-lactate through glycolysis,^20^ we quantified lactate in paired ESCC tissues. Unlike L-lactate, which is abundantly generated by host tumor cells via the Warburg effect, D-lactate is generally considered as a bacteria-derived metabolite in humans. As expected, both L- and D-lactate concentrations were significantly elevated in CATs compared to NATs (Figure 2F​​​ and H). Crucially, intratumor *Lactobacillus* abundance exhibited a strong positive correlation with D-lactate concentrations (*r* = 0.6465, *P* < 0.05, Figure 2I) and a modest positive association with L-lactate (*r* = 0.5557, *P* = 0.05, Figure 2G). These findings collectively support the contribution of viable *Lactobacillus* to intratumor D-lactate accumulation, prompting our subsequent investigations into the mechanistic cascade triggered by microbiota-derived lactate in ESCC.

### *L. reuteri*-derived D-lactate promotes ESCC cell proliferation

**Figure 3.**
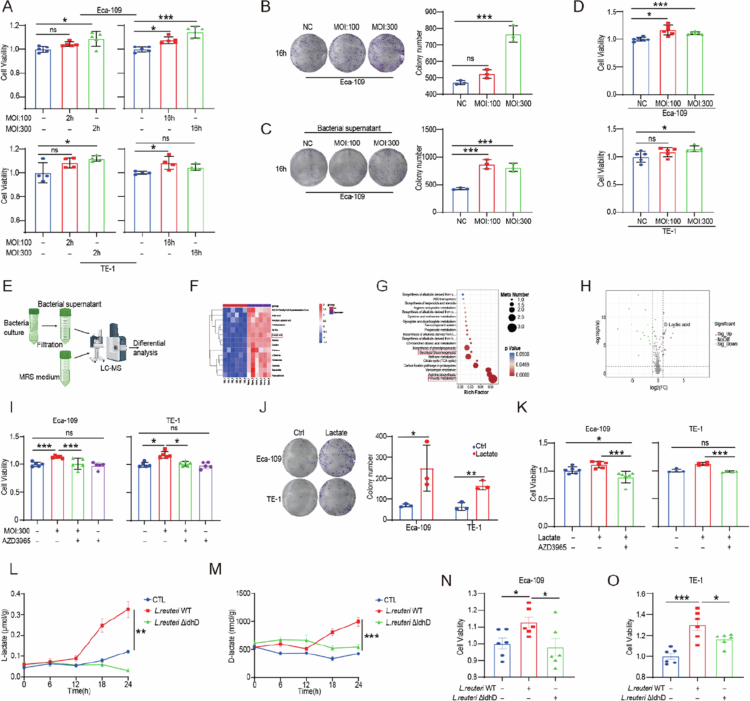
*L. reuteri*-derived D-lactate promotes ESCC cell proliferation. (A) Cell viability after *L. reuteri* treatment measured by CCK-8 assay (*n* = 4–5). (B, C) Representative colony formation images (B) and quantification (C) of Eca-109 cells treated as indicated (*n* = 3). (D) Cell viability of Eca-109 and TE-1 cells treated as indicated (*n* = 5‒6). (E) Workflow for LC–MS analysis of *L. reuteri* culture supernatant. (F) Heatmap showing distinct metabolites between bacterial culture supernatant and uninoculated medium. (G) Pathway enrichment analysis of metabolites enriched in the *L. reuteri* supernatant. (H) Volcano plot showing differentially abundant metabolites between MRS medium and *L. reuteri* culture supernatant. (I) Cell viability of Eca-109 and TE-1 cells treated as indicated; AZD3965 was used to block cellular lactate transport (*n* = 5). (J) Colony formation of Eca-109 and TE-1 cells treated with lactate (*n* = 3). (K) Cell viability of Eca-109 and TE-1 cells treated as indicated (*n* = 4–6). (L-M) Multiline charts showing time-course of intracellular L-lactate and D-lactate levels in tumor cells cocultured with either WT or *ldhD*-deficient *L. reuteri* strains (*n* = 6). (N, O) Cell viability of Eca-109 and TE-1 cells treated as indicated (*n* = 6). Data are presented as means ± SEM. “*n*” represents the number of biologically independent replicates per group. For multiple comparative groups, a one-way ANOVA followed by Tukey's post hoc test was applied. Predefined pairwise comparisons across two conditions were conducted using independent Student's t-tests. For the time-course tracking assays (L, M), statistical significance between groups was evaluated exclusively at the terminal endpoint using the unpaired two-tailed Student's t-test. *P < 0.05, **P < 0.01, ***P < 0.001, ns: not significant. Abbreviations: ESCC, esophageal squamous cell carcinoma; CCK-8, Cell counting kit-8; LC-MS, liquid chromatography- mass spectrometry.

Next, we examined whether tumor-enriched *Lactobacillus* modulates ESCC cell behavior. As *L. reuteri* was preferentially enriched in tumor tissues (Figure 1M) and its role in ESCC remains undefined, we selected this species for subsequent experiments. Co-culture with *L. reuteri* increased the viability of TE-1 and Eca-109 cells in a multiplicity-of-infection (MOI)-and time-dependent manner (Figure 3A), with the pro-proliferative advantage persisting for up to 3 d after treatment (Supplementary Figure S3A-B). *L. reuteri* also enhanced clonogenic growth (Figure 3B). To identify the potent effectors mediating this phenotype, we tested cell-free bacterial culture supernatants. The *L. reuteri* supernatant recapitulated the pro-growth effects in both cell viability and colony formation assays (Figure 3C-D), whereas heat-killed *L. reuteri* failed to stimulate proliferation (Supplementary Figure S3C), indicating that soluble factors released by *L. reuteri* drive the observed effects.

Subsequently, we performed liquid chromatography-mass spectrometry (LC-MS) to profile metabolites in the bacterial supernatant (Figure 3E). Compared with pure culture medium, *L. reuteri* supernatant showed enrichment of multiple metabolites including D-lactate (Figure 3F). KEGG pathway analysis highlighted a profound enrichment in pyruvate metabolism and glycolysis (Figure 3G), and targeted quantification confirmed markedly higher level of D-lactate in the bacterial supernatant (Figure 3H). We therefore hypothesized that D-lactate produced by *L. reuteri* promoted cell proliferation. Indeed, exogenous supplementation revealed that D-lactate, but not L-lactate, promoted ESCC cell viability in a concentration-dependent manner (Supplementary Figure S3D). To further confirm the necessity of lactate cellular uptake, we employed AZD3965, a selective monocarboxylate transporter 1 (MCT1) inhibitor, which effectively diminished intracellular D-lactate accumulation (Supplementary Figure S3E). AZD3965 alone had minimal impact on proliferation but significantly attenuated the pro-proliferative effect of *L. reuteri* supernatant (Figure 3I, Supplementary Figure S3F). Consistently, exogenous sodium racemic lactate (20 mM) stimulated Eca-109 and TE-1 clonogenic growth, and AZD3965 blunted this response (Figure 3J-K).

To further test the causal contribution of *L. reuteri*-derived D-lactate, we generated a mutant strain lacking the D-lactate dehydrogenase gene (*L. reuteri* Δ*ldhD*) by homologous recombination, as described previously^21^ (Supplementary Figure S3G). As expected, compared with wild-type (WT) *L. reuteri*, *ldhD* deficiency resulted in compromised D-lactate production (Supplementary Figure S3H). During real-time coculture tracking, intracellular D-lactate and subsequently L-lactate levels in host tumor cells surged predominantly at 12 hours with WT *L. reuteri*; however, this dynamic intracellular lactate accumulation was markedly abrogated upon coculture with *L. reuteri* ΔldhD (Figure 3L-M). Correspondingly, the *L. reuteri* Δ*ldhD* strain largely diminished the proproliferative effect triggered by the WT bacteria (Figure 3N-O). Collectively, these results support a model in which *L. reuteri*-derived D-lactate directly fuels ESCC tumor cell proliferation.

### D-lactate modifies STAT3 lysine 631 and promotes its nuclear translocation

**Figure 4.**
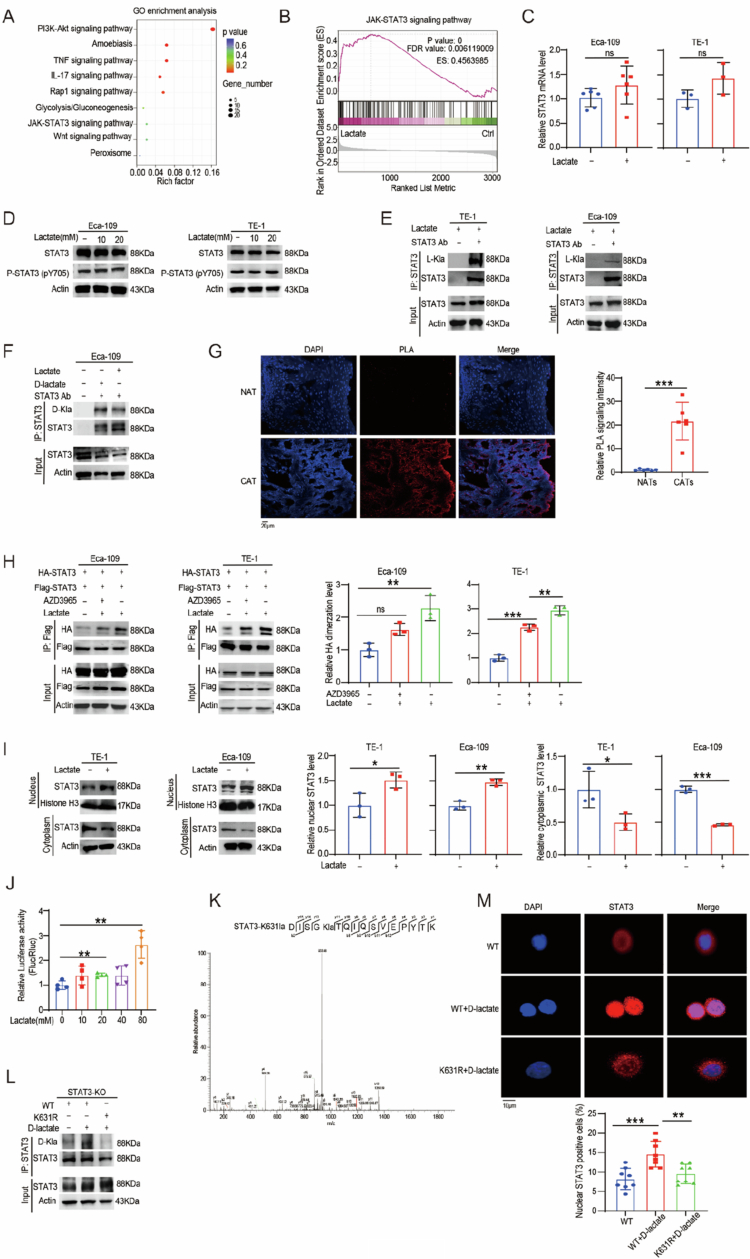
D-lactate modifies STAT3 via lysine 631 lactylation and promotes its nuclear translocation. (A) Gene Ontology (GO) enrichment analysis of differentially expressed lactate-regulated transcripts in Eca-109cells. (B) Gene set enrichment analysis (GSEA) plot showing significant upregulation of the JAK-STAT3 signaling pathway in Eca-109cells treated with lactate. (C) Relative *STAT3* mRNA expression in Eca-109 and TE-1 cells following lactate treatment, measured by RT‒qPCR (*n* = 3 or 6). (D) Immunoblotting showing protein levels of the indicated proteins in Eca-109 and TE-1 cells treated with lactate. (E) Immunoprecipitation (IP) of STAT3 from Eca-109 and TE-1 cells treated with sodium racemic lactate (20 mM, 24 h), followed by immunoblotting with anti-L-lactyllysine antibody. (F) IP of STAT3 from Eca-109 cells treated with sodium racemic lactate or pure D-lactate (20 mM, 24 h), followed by immunoblotting with anti-D-lactyllysine antibody. (G) Duolink^®^ proximity ligation assay (PLA) demonstrating *in situ* interaction signals (red dots) indicating lactylated STAT3 in CATs versus NATs. Nuclei were counterstained with DAPI. Scale bar, 20 μm (*n* = 6). (H) Coimmunoprecipitation showing STAT3 dimerization in cells cotransfected with HA-STAT3 and Flag-STAT3 and treated as indicated (*n* = 3). (I) Immunoblotting of fractionated cytoplasmic and nuclear cell lysates, indicating increased nuclear STAT3 accumulation post-lactate treatment (*n* = 3). (J) STAT3 transcriptional activity measured by dual-luciferase reporter assay following sodium racemic lactate treatment (20–80 mM) in the presence of IL-6 (*n* = 6). (K) High-resolution LC-MS/MS spectral identification of the uniquely modified target peptide, confirming lactylation specifically at the Lysine 631 (K631) residue of STAT3. (L) Validation IP assay confirming the essentiality of K631. CRISPR STAT3-KO Eca-109 cells were reconstituted with wild-type STAT3 or STAT3-K631R mutant plasmids, respectively. (M) Immunofluorescence tracking STAT3 subcellular localization in the reconstituted STAT3-KO cell models, followed by D-lactate treatment for 24 h. Representative images and corresponding quantitative data are displayed. Scale bar, 20 μm (*n* = 6). Data are presented as means ± SEM. “*n*” represents the number of independent *in vitro* biological replicates per condition. Measurements between paired clinical samples utilized the paired two-tailed Student's t-test (G). Multiple comparisons were evaluated using One-way ANOVA followed by Tukey's post-hoc test was applied. Predefined pairwise comparisons were conducted using an unpaired Student's t-tests. *P < 0.05, **P < 0.01, ***P < 0.001, ns: not significant. Abbreviations: CATs, cancer tissues; NATs, normal adjacent tissues; WB, western blot; IP, immunoprecipitation; PLA, proximity ligation assay; KO, knockout.

To define the molecular basis of lactate-driven proliferation, we treated Eca-109 cells with sodium racemic lactate and performed RNA sequencing. Gene Ontology enrichment and gene set enrichment analysis (GSEA) consistently revealed a top-ranked activation of the JAK-STAT3 signaling pathway (Figure 4A-B). Nevertheless, sodium racemic lactate stimulation failed to alter *STAT3* mRNA transcription, total STAT3 protein abundance, or its canonical phosphorylation levels (*P*-STAT3 tyrosine 705) (Figure 4C-D), suggesting a noncanonical mechanism of STAT3 activation.

Recently, lysine lactylation (Kla) driven by cellular lactate accumulation has been reported.^22^ ^,^ ^23^ Therefore, we explored whether lactate could regulate STAT3 via lactylation. Immunoprecipitation (IP) assays confirmed the presence of STAT3 lactylation in both Eca-109 and TE-1 cells (Figure 4E). As mentioned previously, *L. reuteri* is preferentially enriched in CATs and serves as a major source of D-lactate, a stereoisomer typically maintained at minimal levels in baseline mammalian cells. Notably, immunoblotting of immunoprecipitated STAT3 showed that D-lactate, but not L-lactate, markedly increased the anti-D-lactyllysine signal on STAT3 (Figure 4F, Supplementary Figure S3J). *In situ* proximity ligation assays further revealed elevated STAT3 lactylation signals in the epithelium of CATs compared with matched normal adjacent tissues (Figure 4G). Functionally, lactylation markedly potentiated STAT3 homo-dimerization, a structural requisite for activation, which was effectively completely abrogated by AZD3965 treatment (Figure 4H). It is well established that dimerization reshapes STAT3 conformation, driving its nuclear translocation to direct downstream targeted gene expression.^24^ Consistently, sodium racemic lactate promoted STAT3 nuclear accumulation as shown by nuclear/cytoplasmic fractionation immunoblotting (Figure 4I) and immunofluorescence (IF) assays (Supplementary Figure S3I). Moreover, dual-luciferase reporter assays validated that sodium racemic lactate escalated STAT3 transcriptional activity in a concentration-dependent manner (Figure 4J).

To map the lactylation sites, STAT3 was immunoprecipitated from Eca-109 cells and analyzed by LC-MS/MS, which identified lysine 631 (K631) as the D-lactate-responsive lactylation site (Figure 4K). To validate the mechanistic indispensability of K631, we generated a lactylation-deficient STAT3 mutant by substituting K631 with arginine, and established STAT3-knockout (STAT3-KO) Eca-109 cells using the CRISPR/Cas9 (Supplementary Figure S3K). Then, we performed rescue experiments in STAT3-KO cells transfected with either a wild-type STAT3 plasmid or a K631R mutant variant. Subsequent IP assays functionally demonstrated that the K631 point mutation almost entirely abolished D-lactate-induced STAT3 lactylation (Figure 4L). Furthermore, IF tracking assays vividly illustrated that the K631R mutation severely impaired the nuclear translocation ability of STAT3, even upon D-lactate stimulation (Figure 4M). Therefore, these findings verified the mechanistic role of STAT3 K631 lactylation in orchestrating its nuclear translocation and functional hyperactivation.

### D-lactate attenuates RSL3-induced ferroptosis through STAT3 K631 lactylation

**Figure 5.**
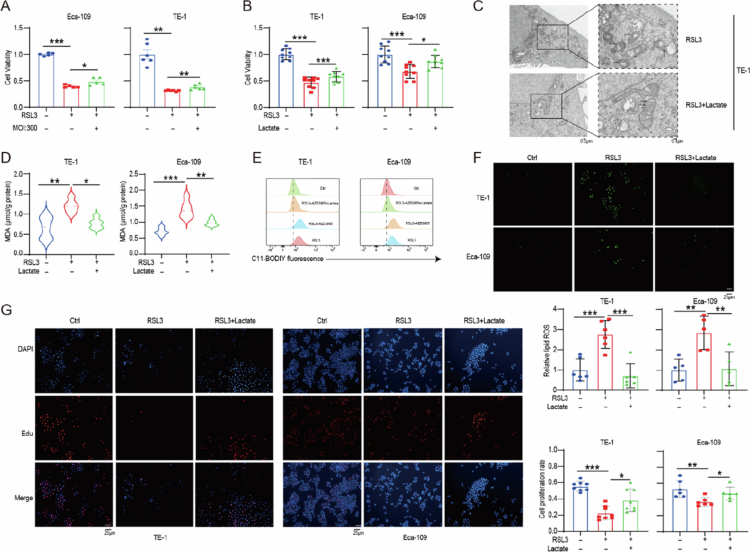
*L. reuteri-*derived D-lactate inhibits ferroptosis in esophageal cancer cells. (A, B) Cell viability of Eca-109 and TE-1 cells treated with cell-free *L. reuteri* conditioned medium (A) or 20 mM sodium racemic lactate for 24 h (B), combined with or without the ferroptosis inducer RSL3 (100 nM), measured by CCK-8 assay (*n* = 5–9). (C) TEM images of TE-1 cells showing morphologic mitochondrial changes (shrunken mitochondria and diminished cristae, black arrows) induced by RSL3, with or without sodium racemic lactate administration. Scale bars from left to right, 0.2 and 0.1 μm. (D) Quantification of intracellular MDA concentrations in TE-1 and Eca-109 cells treated as indicated (*n* = 5–6). (E, F) Evaluation of ROS accumulation utilizing the C11-BODIPY 581/591 fluorescent probe (E) and subsequent microscopy Liperfluo staining (F) in TE-1 and Eca-109 cells treated with RSL3 and/or sodium racemic lactate. Scale bar, 25 μm (*n* = 5–6). (G) Evaluation of cellular DNA synthesis and proliferative capacity utilizing EdU incorporation assays under indicated conditions. Representative fluorescence images and corresponding quantifications of EdU-positive cells are shown. Scale bar, 25 μm (*n* = 6–8). Data are presented as means ± SEM. “n” represents independent cell samples or representative fields, as indicated. Statistical significance among groups in panels was determined universally using one-way ANOVA followed by Tukey's post hoc test. Targeted pairwise comparisons were determined utilizing individual unpaired two-tailed Student's t-tests. *P < 0.05, **P < 0.01, ***P < 0.001. Abbreviations: TEM, transmission electron microscope; MDA, malondialdehyde; ROS, reactive oxygen species.

To explore the mechanism underlying the pro-proliferative effect, we first investigated whether lactate could rescue ESCC cells from specific modes of programmed cell death. As shown in Supplementary Figure S3L, D-lactate failed to protect cells from pyroptosis or apoptosis. Given the emerging therapeutic interest in ferroptosis in ESCC^25^ ^,^ ^26^ and the unclear role of STAT3 in this context, we next examined ferroptotic sensitivity. Strikingly, live *L. reuteri* attenuated RSL3-induced ferroptosis in Eca-109 cells, whereas the *ldhD*-deficient mutant *L. reuteri* failed to confer such protection (Supplementary Figure S4A), indicating that bacteria-derived D-lactate is the functional effector. Consistently, cell-free *L. reuteri* conditioned medium protected both Eca-109 and TE-1 cells from RSL3-triggered ferroptosis (Figure 5A). Moreover, sodium racemic lactate partially rescued RSL3-treated cells, increasing viability by approximately 20% compared with RSL3 alone (Figure 5B). TEM showed that TE-1 cells treated with RSL3 exhibited shrunken mitochondria and decreased mitochondrial cristae density, while sodium racemic lactate markedly ameliorated these mitochondrial lesions (Figure 5C). Biochemically, sodium racemic lactate effectively suppressed intracellular malondialdehyde (MDA) levels (Figure 5D) and quenched lipid ROS bursts, measured by C11-BODIPY fluorescent tracing utilizing flow cytometry and microscopy Liperfluo staining (Figure 5E-F). In line with these anti-ferroptotic effects, EdU incorporation showed that sodium racemic lactate restored proliferative activity compared with RSL3 alone (Figure 5G).

As mentioned above, D-lactate induces STAT3 lactylation at K631 residue. To verify the essential role of STAT3 lactylation in regulating ferroptosis, we treated STAT3-KO Eca-109 cells with RSL3 and reconstituted with either wild-type STAT3 plasmid or lactylation-defective STAT3 K631R mutant plasmid. Crucially, expression of the STAT3-K631R mutant largely abolished the ferroptosis-inhibitory function of STAT3 (Supplementary Figure S4B). Together, these results support a model in which D-lactate rescues ESCC cell lines from ferroptosis through a STAT3 K631 lactylation-dependent mechanism.

### Correlation of intratumor *Lactobacillus* abundance with GPX4/FTH1 expression in ESCC

**Figure 6.**
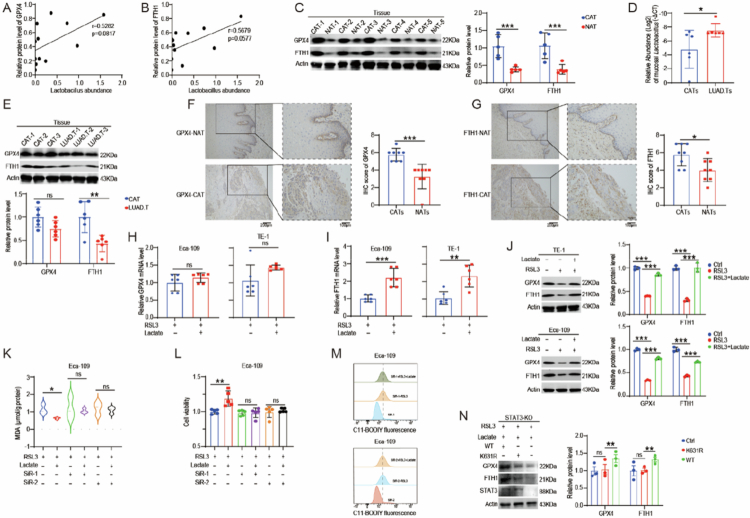
*Lactobacillus-*derived D-lactate metabolically upregulates GPX4/FTH1 in a STAT3 K631 lactylation-dependent manner. (A, B) Spearman's rank correlation analyzes illuminating the relationship between intratumor *Lactobacillus* abundance and protein levels of GPX4 (A) or FTH1(B) in tissue samples. (C) GPX4 and FTH1 protein levels in paired CATs and NATs measured by western blotting; quantification is shown on the right. Paired two-tailed Student's t-test (*n* = 5 pairs). (D) qPCR quantification of *Lactobacillus* abundance in ESCC CATs and lung adenocarcinoma (LUAD) tumor tissues. Mann‒Whitney U test (*n* = 3, technical duplicates). (E) GPX4 and FTH1 protein levels in ESCC CATs and LUAD tumor tissues were measured by western blotting, with quantification shown below. (*n* = 3, technical duplicates). (F, G) Representative IHC images of CTAs and NATs probing GPX4 and FTH1 expression. Scale bars, 200 μm and 100 μm from left to right, respectively. Semi-quantitative IHC scores are shown. Paired two-tailed Student's t-test (*n* = 8). (H, I) Relative *GPX4* and *FTH1* mRNA expression in Eca-109 and TE-1 cells treated as indicated (*n* = 6). (J) GPX4 and FTH1protein levels in Eca-109 and TE-1 cells treated as indicated; quantification is shown (*n* = 3). (K) Intracellular MDA concentrations in Eca-109 cells treated as indicated (*n* = 3). (L) Cell viability of Eca-109 cells treated as indicated (*n* = 5). (M) Cellular lipid ROS levels in Eca-109 cells treated as indicated. (N) GPX4 and FTH1 protein levels in STAT3-KO Eca-109 cells reconstituted with wild-type or STAT3 K631R plasmid and treated as indicated; quantification is shown (*n* = 3). Data are presented as means ± SEM. “n” represents independent cell samples, representative fields, tissue samples or protein bands, as indicated. Independent sets representing two groups utilized the unpaired two-tailed Student's t-test, whilst models spanning three or more independent conditions sequentially employed one-way ANOVA followed by Tukey's post hoc test. Targeted statistical significance among specific pairs of interest was determined utilizing individual unpaired two-tailed Student's t-tests. *P < 0.05, **P < 0.01, ***P < 0.001, ns: not significant. Abbreviations: CATs, cancer tissues; NATs, normal adjacent tissues; ESCC, esophageal squamous cell carcinoma; LUAD, lung adenocarcinoma; WB, Western blot; qPCR, quantitative PCR; IHC, immunohistochemistry; MDA, malondialdehyde; ROS, reactive oxygen species.

STAT3 has been demonstrated to inhibit ferroptosis by regulating antiferroptotic proteins, including glutathione peroxidase 4 (GPX4) and ferritin heavy chain 1 (FTH1), in gastric cancer cells.^27^ To determine whether this axis operates in ESCC, we assessed GPX4 and FTH1 after STAT3 knockdown. STAT3 knockdown dramatically downregulated GPX4 and FTH1 protein levels in both Eca-109 and TE-1 cells (Supplementary Figure S4C-D), suggesting that STAT3 contributes to maintaining GPX4/FTH1 expression in ESCC cells. To determine whether this antiferroptotic axis is clinically driven by the local microbiota, we then examined clinical ESCC specimens. In CATs, intratumor *Lactobacillus* abundance exhibited a moderate positive correlation with the protein levels of both GPX4 and FTH1. While these associations marginally fell short of strict statistical significance (*P* < 0.05), they reflected a pronounced positive trend (GPX4: *r* = 0.5282, *P* = 0.0817; FTH1: *r* = 0.5679, *P* = 0.0577) (Figure 6A-B). Consistently, target validation in paired clinical tissues demonstrated that both GPX4 and FTH1 were significantly overexpressed in CATs compared to NATs, as evidenced by immunoblotting (Figure 6C) and corroborated by immunohistochemistry (Figure 6F-G). To further contextualize these findings, we performed parallel analyzes in lung adenocarcinoma (LUAD). In TCGA-LUAD, GPX4 expression did not differ between tumor and adjacent normal tissues, whereas FTH1 was higher in adjacent normal tissues (Supplementary Figure S5). In an independent set of paired LUAD samples (*n* = 3), qPCR indicated lower *Lactobacillus* abundance in LUAD tumors compared with ESCC tumors (Figure 6D), but *Lactobacillus* levels in LUAD tumors and matched normal tissues were almost identical (Supplementary Figure S6A). Western blotting further confirmed lower GPX4 and FTH1 levels in LUAD tumors compared with CATs (Figure 6E). Within LUAD pairs, GPX4 levels were comparable, while FTH1 was higher in LUAD normal tissues (Supplementary Figure S6B). These exploratory comparative data suggest that the *Lactobacillus*-GPX4/FTH1 relationship may be more prominent in ESCC than in LUAD.

Given the positive association between *Lactobacillus* abundance and lactate concentrations, we further explored relationships between D-lactate and ferroptosis regulators. Interestingly, D-lactate positively correlated with GPX4 protein levels (Supplementary Figure S4E-F). STAT3 expression was also higher in CATs than in NATs (Supplementary Figure S4I). Clinically, GPX4 expression was associated with pathological stage, whereas the association for FTH1 was not obvious (Supplementary Figure S4G). Kaplan–Meier analyzes using TCGA and GTEx databases indicated that higher expression levels of GPX4 and FTH1 were associated with shorter survival in ESCC (Supplementary Figure S4H). Together, these results suggest a positive relationship between intratumor *Lactobacillus* abundance and GPX4/FTH1 expression in ESCC, supporting our focus on ferroptosis regulation in this disease.

### STAT3 K631 lactylation modulates GPX4/FTH1 accumulation and ferroptosis resistance

Next, we delineated the precise molecular cascade linking lactate to ferroptosis resistance. Interestingly, exogenous sodium racemic lactate treatment did not significantly alter *GPX4* mRNA abundance and only modestly increased *FTH1* transcripts (Figure 6H-I). Despite modest transcript changes, sodium racemic lactate markedly increased GPX4 and FTH1 protein levels (Figure 6J). Notably, D-lactate (the primary *L. reuteri* metabolite) was specifically potent in driving this protein accumulation compared to L-lactate (Supplementary Figure S6C), suggesting post-transcriptional or translational regulatory mechanisms contributed by D-lactate.

To confirm the indispensable role of the STAT3 signaling node, we performed specific knockdown experiments. Sodium racemic lactate reduced MDA levels; this reduction was abolished in cells transfected with STAT3-targeting siRNA (Figure 6K). Consistently, STAT3 knockdown diminished lactate-mediated protection from RSL3-induced ferroptosis in Eca-109 cells (Figure 6L) and prevented the lactate-associated reduction in lipid peroxidation (Figure 6M). To test whether STAT3 K631 lactylation is essential, we performed rescue experiments in STAT3-KO cells transfected with either a wild-type STAT3 plasmid or a K631R mutant variant. Notably, sodium racemic lactate treatment specifically increased GPX4 and FTH1 protein levels only in cells reconstituted with wild-type STAT3, but not in those expressing STAT3 K631R (Figure 6N). Together, these results provide definitive evidence that *Lactobacillus*-derived D-lactate metabolically programs ESCC cells against ferroptosis dependently through STAT3 K631 lactylation-mediated GPX4/FTH1 upregulation.

### *L. reuteri*-derived D-lactate dictates ESCC tumor growth *in vivo* via the STAT3-GPX4/FTH1 axis

**Figure 7.**
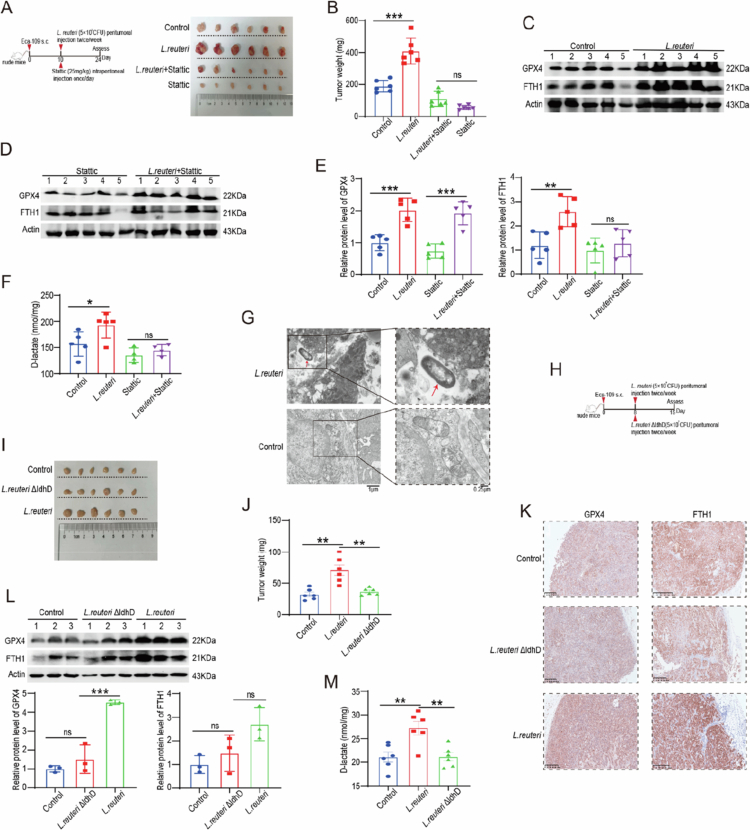
*L. reuteri* promotes *in vivo* ESCC tumor growth through the D-lactate-STAT3-ferroptosis signaling pathway. (A) Schematic of the xenograft experimental design (left) and representative gross images of resected tumors (right) from Eca-109 xenograft-bearing nude mice. Mice were treated with peritumoral *L. reuteri* or PBS twice/week and intraperitoneal Stattic or PBS once/day, as indicated (*n* = 6). (B) Tumor weights from mice in (A) (*n* = 6). (C, D) GPX4 and FTH1 protein levels in tumor tissues from the indicated groups. (E) Quantification of GPX4 and FTH1 protein levels in tumor tissues (*n* = 5). (F) D-lactate concentrations in tumor tissues from mice treated as indicated (*n* = 4–5). (G) TEM images of *L. reuteri* in tumor tissues. Red arrows denote intact bacteria. Scale bars from left to right, 1 and 0.25 μm. (H) Schematic of the xenograft experiment using wild-type *L. reuteri* or *L. reuteri ΔldhD* delivered by peritumoral injection twice weekly; mice were sacrificed on day 15. (I) Representative image of tumors from mice receiving wild-type *L. reuteri* or *L. reuteri ΔldhD* (*n* = 6). (J) Tumor weights from mice in (I) (*n* = 6). (K) Representative IHC images of tumor tissues showing GPX4 and FTH1 expression in the indicated groups. Scale bar, 250 μm. (L) GPX4 and FTH1 protein levels in tumor tissues; quantification is shown (*n* = 3). (M) D-lactate concentrations in tumor tissues (*n* = 6). Data are presented as means ± SEM. “n” represents the number of tumor samples or protein bands in each group. Multigroup *in vivo* datasets were evaluated globally utilizing one-way ANOVA coupled with Tukey's post-hoc test. Analyzes strictly comprising two independent experimental *in vivo* animal arms were assessed robustly using the unpaired two-tailed Student's t-test. *P < 0.05, **P < 0.01, ***P < 0.001, ns: not significant. Abbreviations: s.c., subcutaneous inoculation; TEM, transmission electron microscope; IHC, immunohistochemistry.

We next examined this pathway *in vivo* using an Eca-109 xenograft model. Compared with mock-treated control mice, tumor volumes in the group receiving *L. reuteri* peritumoral injection were significantly larger (Figure 7A). In contrast, pharmacological inhibition of STAT3 with Stattic reduced tumor growth and attenuated the tumorigenicity of *L. reuteri* (Figure 7A-B). It was observed that *L. reuteri* promoted GPX4 and FTH1 expression, whereas Stattic reduced their expression (Figure 7C-D). Notably, *L. reuteri* did not restore FTH1 levels suppressed by Stattic (Figure 7E), elegantly demonstrating that downstream STAT3 structural signaling is the indispensable cellular node bridging bacterial D-lactate to ferroptosis resistance. In addition, D-lactate levels were highest in tumors from *L. reuteri*-treated mice and were slightly elevated when *L. reuteri* was administered together with Stattic (Figure 7F). Furthermore, successful colonization of the administered bacteria within the tumor parenchyma was explicitly confirmed via TEM, with intact bacilli observed inside the tumor tissues (Figure 7G).

In addition, we evaluated the protumor activity of *L. reuteri* Δ*ldhD* to better understand the role of *L. reuteri*-derived D-lactate (Figure 7H). Compared to the wild-type strain, the mutant strain led to significantly reduced tumor weights and volumes (Figure 7I-J). *L. reuteri* Δ*ldhD* weakly promoted the protein expressions of GPX4 and FTH1 compared with wild-type *L. reuteri* (Figure 7K-L) and substantially reduced intratumor D-lactate levels (Figure 7M). In summary, these data support our hypothesis that *L. reuteri* in tumor tissues promotes lactate-dependent ferroptosis resistance via STAT3 K631 lactylation and downstream GPX4/FTH1, thereby facilitating ESCC tumor growth (Figure S7).

## Discussion

The study comprehensively analyzed 102 multiregional tissue samples from 27 patients with ESCC and identified a consistent enrichment of *Lactobacillus*, particularly *L. reuteri*, in CATs. Supported by rigorous negative controls and orthogonal validations (FISH, TEM, and culture) to mitigate low-biomass profiling biases, our findings extend beyond describing a tumor-associated microbial signature. We delineate a mechanistic axis wherein *L. reuteri*–derived D-lactate induces site-specific STAT3 K631 lactylation, promoting its dimerization and nuclear accumulation, thereby upregulating the ferroptosis suppressors GPX4 and FTH1. Our reciprocal genetic strategy, utilizing both the *L. reuteri ΔldhD* strain and the lactylation-deficient STAT3 K631R host cells, together with *in vivo* xenograft validation, unequivocally demonstrates a causal link between microbiota-derived D-lactate and the reprogramming of tumor stress responses.

A fundamental conceptual advance of our study is distinguishing the biological role of bacterial D-lactate from host-derived L-lactate. While prevailing paradigms emphasize the effects of massive host L-lactate production on acidic immunosuppression,^28^ our findings highlight D-lactate as an exceedingly potent signaling metabolite. Because D-lactate is present at minimally low levels in mammalian metabolism, its bacterial origin constitutes a specific microbe-to-host signal. Intracellular transport of bacteria-derived D-lactate is likely facilitated by MCT1, the classic proton-coupled lactate transporter in ESCC (Figure S3D). Once inside the cytosol, this microbial metabolite directly drives nonhistone lysine lactylation. While previous work has shown that STAT3 can be L-lactylated in hepatocellular carcinoma,^22^ our data extend this biology by linking microbe-derived D-lactate specifically to the STAT3 K631 residue. Structurally, K631 lies within the SH2 domain essential for dimerization,^24^ ^,^ ^29^ providing a mechanistic rationale for hyperactivated STAT3 signaling. D-lactate did not increase the overall abundance of Y705-phosphorylated STAT3, K631 lactylation may enhance the dimerization competence and nuclear retention of the pre-existing phosphorylated STAT3 pool, consistent with the location of K631 within the SH2 domain. Although analytical challenges in mass spectrometry remain regarding the complete discrimination of lactylation from chemically related acylations,^30^ our rescue experiments provide robust functional evidence that D-lactate contributes to this lactylation-dependent regulatory mechanism.

Regarding the ecological origin of these tumor-resident bacteria, our data support a niche-specific adaptation model. *Lactobacillus* in the esophagus likely originates from the continuous transit of oral flora, gastric reflux, or dietary intake.^3^ ^,^ ^4^ The structural collapse of the mucosal barrier, hallmarks of ESCC progression and ulceration, facilitates direct luminal translocation into the tumor bed. Once colonized, the inherently hypoxic and acidic tumor milieu selectively favors acid-tolerant taxa like *Lactobacillus*. This explains the seeming abrupt shift of *Lactobacillus* from a “beneficial commensal” in healthy guts to a “pro-tumoral factor” in ESCC, emphasizing that microbial function is strictly sculpted by the local microenvironment.

This context-dependent nature of microbiota-host interactions challenges the simplistic “beneficial versus harmful” classification of probiotics in oncology. While *L.reuteri* enhances immunotherapy efficacy via indole-3-aldehyde in melanoma^31^ and suppresses colon cancer initiation,^32^ recent evidence reveals its darker side: gut indole-producing *L. reuteri* can aggressively promote pancreatic ductal adenocarcinoma via immunosuppressive macrophages.^33^ Furthermore, tumor-intrinsic STAT3 hyperactivation is a well-established driver of immunosuppression (e.g., via IL-6/IL-10 secretion),^34^ and overcoming ferroptosis prevents the release of immunogenic damage-associated molecular patterns (DAMPs).^35^ Beyond these tumor-intrinsic signaling cascade, *Lactobacillus* species and the associated accumulation of environmental lactates exert direct immunomodulatory effects. Intratumor lactate is known to functionally induce PD-L1 expression, recruit immunosuppressive cells, and paralyze cytotoxic lymphocyte infiltration.^36^ Thus, in ESCC, *Lactobacillus* exerts a deleterious effect, strongly echoing recent preclinical cautions that unselected probiotic supplementation may impair antitumour immunity or accelerate progression.^37-39^

Our study presents several notable strengths and limitations. Methodologically, by sampling 102 multiregional blocks from 27 patients, we uncovered substantial intrapatient spatial heterogeneity in bacterial communities. This highlights that single-biopsy approaches may underrepresent true intratumor microbial diversity, underscoring the necessity of multiregional sampling in future biomarker designs. Owing to the small clinical cohort, limited number of tissue samples available for D-lactate quantification, and unadjusted confounders such as proton pump inhibitor use and diet, these exploratory findings require validation in larger multicenter cohorts, despite consistent results from *in virto* and *in vivo* experiments. Second, while our *in vivo* peritumoral injection serves as an effective proof-of-concept, it does not fully recapitulate natural colonization; future studies employing oral gavage or spontaneous models are warranted to dissect natural dissemination routes. Finally, recognizing that matched NATs may possess preneoplastic alterations due to field cancerization, strict comparisons against authentic healthy esophageal mucosa will further refine ESCC-specific microbial signals.

In conclusion, our work defines a previously underappreciated tumor-resident microbe–metabolite–host signaling axis. By establishing *Lactobacillus*-derived D-lactate as a mechanistic driver of STAT3 K631 lactylation and ferroptosis escape, we provide a compelling framework that extends beyond standard metabolic paradigms. These findings inform the development of novel therapeutic strategies targeting STAT3 lactylation and/or ferroptosis sensitization in ESCC, while cautioning against the indiscriminate clinical use of probiotics in this vulnerable population.

## Supplementary Material

Supplementary Table S3.xlsx Supplementary Table S3.xlsx

Supplementary Table S4.xlsx Supplementary Table S4.xlsx

Supplemental_material.docx Supplemental_material.docx

Supplementary Table S2.xlsx Supplementary Table S2.xlsx

Supplementary Table S1.xlsx Supplementary Table S1.xlsx

## Data Availability

Raw data of 16S rRNA sequencing and RNA sequencing had been uploaded to the National Center for Biotechnology Information (NCBI) Sequence Read Archive database under the following respective accession numbers: PRJNA972350 and PRJNA971160. The TCGA esophageal cancer datasets (https://portal.gdc.cancer.gov) and Timer 2.0 dataset (http://timer.comp-genomics.org/) were analyzed in this paper, and the Kaplan–Meier analysis of ESCC patients were generated on the GEPIA 2 website (gepia2.cancer-pku.cn). The datasets generated during the current study are available from the corresponding author on reasonable request.
