# Supplementary material for: Intratumor Lactobacillus drives ferroptosis resistance via D-lactate-STAT3 K631 lactylation in esophageal squamous cell carcinoma
Source: Gut Microbes. 2026 Jun 11;18(1):2685912. doi: 10.1080/19490976.2026.2685912 (PMC13274160; doi:10.1080/19490976.2026.2685912)
Supplement: Supplemental_material.docx [file KGMI_A_2685912_SM4806.docx]

**Intratumor *Lactobacillus* drives ferroptosis resistance via D-lactate-STAT3 K631 lactylation in esophageal squamous cell carcinoma**

Dong Wang^1,2, †^, Heng Lu^3, †^, Weiguang Li^1, †^, Liuying Li ^3, †^, Xuesen Xu^1,2^, Huimin Zhou^2^, Hui Tao^3^, Yizhen Li^2^, Wei Wei^4^, Kunpeng Wu^5^, Yihan Wang^2^, Run Li^2^, Yuzhong Li^2^, Weiyan Yao^1,*^, Yanwen Chen^1,*^, Fangyu Wang^3,^^*^ , Lei Ye^1,*^

**Affiliations**

^1^**Department of Gastroenterology**, Ruijin Hospital, Shanghai Jiao Tong University School of Medicine, Shanghai, P. R. China

^2^ **State Key Laboratory of Natural Medicines**, School of Pharmacy, China Pharmaceutical University, Nanjing, Jiangsu, P. R. China

^3^ **Department of Gastroenterology and Hepatology**, Jinling Hospital, Affiliated Hospital of Medical School, Nanjing University, Nanjing, Jiangsu, P. R. China

^4^ **Department of Esophageal Surgery**, Department of Thoracic Surgery, Nanjing Drum Tower Hospital, The Affiliated Hospital of Nanjing University Medical School, Nanjing, Jiangsu, P. R. China

^5^ **Department of Thoracic Surgery**, Nanjing Lishui People’s Hospital, Zhongda Hospital Lishui Branch, Southeast University, Nanjing, Jiangsu, P. R. China

^†^ Dong Wang, Heng Lu, Weiguang Li and Liuying Li contributed equally.

^*^**Correspondence:**

Lei Ye ([njuyelei@163.com](mailto:njuyelei@163.com))

Department of Gastroenterology, Ruijin Hospital, Shanghai Jiao Tong University School of

Medicine, Shanghai, P. R. China

Fangyu Wang ([wangfy65@nju.edu.cn](mailto:wangfy65@nju.edu.cn))

Department of Gastroenterology and Hepatology, Jinling Hospital, Affiliated Hospital of Medical School, Nanjing University, Nanjing, Jiangsu, P. R. China

Yanwen Chen ([cywrjh@126.com](mailto:cywrjh@126.com))

Department of Gastroenterology, Ruijin Hospital, Shanghai Jiao Tong University School of Medicine, Shanghai, P. R. China

Weiyan Yao (ywy11491@rjh.com.cn)

Department of Gastroenterology, Ruijin Hospital, Shanghai Jiao Tong University School of Medicine, Shanghai, P. R. China

**Methods**

**16S rRNA and** ***Lactobacillus* fluorescence *in situ* hybridization (FISH) Assay**

Bacterial colonization in CATs and paired NATs was assessed by fluorescence in situ hybridization (FISH). A universal bacterial probe targeting the 16S rRNA gene (EUB338; sequence: GCTGCCTCCCGTAGGAGT) labeled with the fluorophore Cy3 was used to detect total bacteria. A *Lactobacillus*-specific probe (sequence: AGCTTCAATCTTCAGGAT) labelled with the fluorophore FAM was used to detect *Lactobacillus*. Specificity was supported by no-probe/RNase controls and by parallel staining in matched NATs. The FISH protocol followed that described by Nejman et al[1].

**Immunohistochemistry (IHC)**

Tissues were fixed in 4% formalin in PBS and embedded in paraffin (JB-P5, Junjie Electronic Company, Wuhan, P. R. China). Paraffin sections (2 μm) were cut and processed for IHC staining using standard deparaffinization and rehydration procedures. Antigen retrieval was performed in boiling citrate buffer for 10 min. Sections were blocked with 5% normal goat serum supplemented with 0.1% Triton X-100 (P0096 , Beyotime, Shanghai, P. R. China) and 3% H_2_O_2_ in PBS for 60 min at room temperature, followed by incubation with primary antibodies (anti-LPS antibody, 1:100 , Hycult Biotech, HM6011; anti-LTA antibody, 1:100 , Hycult Biotech, HM2048; anti-GPX4 antibody, 1:200 , Abcam, ab125066; anti-FTH1 antibody, 1:200 , Abcam, ab75972; anti-STAT3 antibody, 1:500 , Abcam, ab119352) at 4℃ overnight. Signals were developed using HRP- conjugated secondary regents with DAB detection. Nuclei were counterstained with DAPI (0100-20, Southern Biotech, Alabama, USA). Slides were visualized using Nikon microscopy. Protein expression was semi-quantified based on staining extent and intensity as reported previously[2].

**Sample Preparation for Transmission Electron Microscopy (TEM)**

For cell samples, pellets were fixed with 3% glutaraldehyde in 0.1 M phosphate buffer (pH 7.4), followed by fixation with 1% osmic tetroxide. Samples were dehydrated and embedded, and ultrathin sections (60–80 nm) were prepared and stained with uranyl acetate and lead nitrate prior to imaging using a transmission electron microscope (HITACHI, HT7800, Tokyo, Japan). For fresh tissues, a sharp blade was used to cut and harvest fresh tissue blocks within 1-3 min. Tissue pieces (≤ 1 mm^3^) were immediately transferred into TEM fixative for further processing. Procedures followed published methods[3].

**Plasmids, siRNA, and transfection**

Flag- and HA-tagged STAT3 plasmids were provided by Dr. Yi Jiang (Renji Hospital, Shanghai, P. R. China). The STAT3 K631R plasmid was constructed by Sangon Biotech (Shanghai, P. R. China). Plasmids were transfected using Lipofectamine 3000 (L3000-015, Invitrogen, California, USA) for 48-72 h. Where indicated, cells were treated with 20 mM sodium racemic lactate or 20 nM AZD3965 for the final 24 h. For RNA interference, ESCC cells at ~ 70% confluence in 6-well plates were transfected with STAT3 siRNA or control siRNA using Lipofectamine 3000, and harvested at 48 or 72 h for subsequent experiments. STAT3 siRNA sequences were adopted from previously published methods[4] and synthesized by Sangon Biotech (Shanghai, P. R. China).

**Quantification of *Lactobacillus* and *L. reuteri*** **abundance**

Tissue DNA was extracted from mouse tumor tissues (ESCC, colorectal cancer, or hepatocellular carcinoma) and human tumor tissues (ESCC or lung adenocarcinoma) using the SPINeasy^TM^ DNA Kit for Tissue (116558050, MPBio, California, USA). For each sample, 160 ng of tissue DNA was analyzed by quantitative PCR to estimate *Lactobacillus* abundance.

The abundance of *L. reuteri* in ESCC tissues was further analyzed by qPCR. Relative abundance of *Lactobacillus* and *L. reuteri* was normalized to total bacteria load using the 2^-ΔCt^ method (ΔCt: average of Ct value of *Lactobacillus* / *L. reuteri* - average Ct value of total bacteria)[5].

**Generation of the** ***ldhD*-deficient *L. reuteri* strain**

A *ldhD*-deficient *L. reuteri* strain was generated as reported previously[6]. Briefly, approximately 1.0 kb regions upstream and downstream of *ldhD* were amplified using primer pairs *ldhD*-LABK-F/*ldhD*-up-R and ldhD-down-F/ldhD-LABK-R, respectively, and cloned into pNZ5319 containing an erythromycin-resistance cassette. The recombinant plasmid was electroporated into *L. reuteri*. Single-crossover integrants were selected on erythromycin-containing MRS agar, followed by culture in erythromycin-free MRS to facilitate double-crossover recombination. Mutants were identified by PCR and Sanger sequencing. The primers used in the experiment are listed in **Supplementary Table S1.**

**RNA sequencing**

Total RNA was extracted with TRIzol reagent (Invitrogen, Carlsbad, CA, USA) from Eca-109 cells treated with 20 mM lactate (HY-B2227B, MCE, New Jersey, USA) or phosphate-buffered saline (PBS) for 24 h. Library preparation and paired-end sequencing (PE150) were performed on an Illumina Novaseq™ 6000 platform (LC-Bio Technology Co., Ltd., Hangzhou, P. R. China) following the vendor's recommended protocol.

Reads were quality controlled using fastp and aligned to the human reference genome (GRCh38) using HISAT2. Transcript abundance was quantified as FPKM. Differentially expressed mRNAs were defined using fold change > 2 or < 0.5 and a parametric model-comparison test (p value < 0.05) via the R packages edgeR and DESeq2. Gene Ontology (GO) and KEGG pathway enrichment analyses were subsequently performed.

**Untargeted** **metabolomic analysis of** ***L. reuteri***

*L. reuteri* strain (ATCC 23272) was cultured in MRS broth at 37℃ in sealed tubes under aerobic conditions. Culture supernatants were collected by removing cells and filtering through a 0.22 μm membrane; uninoculated medium was used as a control. Metabolites were extracted using 50% methanol buffer and analyzed by LC-MS/MS using a TripleTOF 5600plus high-resolution tandem mass spectrometer (SCIEX, UK) in both positive and negative ion modes. Raw data were converted to mzXML and processed using XCMS, CAMERA and metaX. Metabolites were annotated using KEGG and HMDB, with further validation using an in-house fragment spectrum library. Peak intensity data were preprocessed using metaX; PCA was performed for outlier detection and batch-effect evaluation. QC -based robust LOESS signal correction was applied. Student’s t-tests were conducted to compare metabolite concentrations between groups, with FDR adjustment for multiple tests. Supervised PLS-DA was conducted through metaX to discriminate the different variables between groups.

**Identification of STAT3 lactylation sites by LC-MS/MS**

Cells were harvested and proteins separated by SDS-PAGE. Following Coomassie staining, gel bands corresponding to the molecular weight of STAT3 were excised and cut into 1 cubic millimeter pieces. Gel pieces were destained in 50% ethanol overnight and washed twice in 50mM NH4HCO_3_. Proteins were digested with trypsin (final concentration 20 ng/μL in 50mM NH4HCO_3_ at 37℃ overnight. Peptides were extracted by dehydration in 100% acetonitrile (twice), pooled and vacuum-dried. Peptides were reconstituted in 50mM NH4HCO_3,_ reduced with 10 mM DTT (37℃, 60min), and alkylated with iodoacetamide (50mM, room temperature, 45min, dark). Peptides were desalted with C18 ZipTips (Millipore).

The tryptic peptides were dissolved in solvent A, directly loaded onto a home-made reversed-phase analytical column (25-cm length, 100 μm i.d.). The mobile phase consisted of solvent A (0.1% formic acid, 2% acetonitrile/in water) and solvent B (0.1% formic acid, 90% acetonitrile/in water). Peptides were separated with the following gradient: 0-16 min，6%-25%B；16-22 min，25%-35%B；22-26 min，35%-80%B；26-30 min，80%-80%B, and all at a constant flow rate of 450 nl/minon an EASY-nLC 1000 UPLC system (ThermoFisher Scientific). The separated peptides were analyzed in Orbitrap Exploris 480 with a nano-electrospray ion source. The electrospray voltage applied was 2100 V. The full MS scan resolution was set to 60000 for a scan range of 350-1800 m/z. MS/MS scans were acquired at 15,000 resolution (fixed first mass 100 m/z), with up to 20 precursors selected per cycle and dynamic exclusion of 15 s. HCD was performed at NCE 28%, with AGC target 50%, intensity threshold 5000 ions/s and maximum injection time 200 ms.

MS/MS data were processed using Proteome Discoverer (v.2.4). Spectra were searched against the STAT3 sequence. Trypsin/P was specified as the cleavage enzyme allowing up to four missed cleavages. Precursor mass tolerance was 10 ppm and fragment mass tolerance 0.02 Da. Carbamidomethylation of cysteine was set as a fixed modification; N‑terminal acetylation, methionine oxidation and lysine lactylation, +72.0211 Da, was set as variable modifications. Identifications required peptide score >20 and high confidence.

**CRISPR/Cas9-mediated STAT3 knockout**

STAT3 knockout in Eca-109 cells was generated using CRISPR/Cas9. sgRNA was cloned into the gRNA-P2A-spCas9 vector and transfected into Eca-109 cell line with polybrene. Puromycin selection was applied 48 h after transfection. Single-cell clones were obtained by serial dilution and expanded. Genomic DNA was extracted and PCR products were ligated into a T-vector for sequencing. Knockout efficiency was evaluated by western blotting. sgRNA targeting STAT3: cattcgactcttgcaggaagcgg.

**Cell-based functional assays**

To investigate the protective effect of lactate on ferroptosis, cells were seeded into 96-well plates and treated with 100 nM RSL3 (HY-100218A, MCE, New Jersey, USA) with or without 20 mM sodium racemic lactate, or 20 nM AZD3965 (HY-12750, MCE, New Jersey, USA) for 24 h. Viability was assessed using CCK-8 (CK04, DOJINDO, Kumamoto, Japan) and absorbance measured at 450 nm.

For bacterial exposure experiments, cells were seeded in 6-well plates, treated with *L. reuteri* at specified MOIs, and subsequently transferred to 96-well plates for viability assessment.  Where indicated, bacterial culture supernatants collected at the specified MOIs were added to cell culture medium after removal of bacterial pellets by centrifugation. Heat‑killed bacteria were generated by incubating bacterial suspensions (OD_600_=1) at 65°C for 15 min prior to co-culture. To examine the effect of lactate on cell colony formation, 24 hours prior, TE-1 and Eca-109 cells were seeded into 6-well plates at 1000 cells/well. Then, sodium racemic lactate (20 mM) was added for 24 h. Media were replaced every 3 days. Approximately 10 days later, colonies were fixed with 4% paraformaldehyde and stained with crystal violet.

EdU assays were performed using the Cell-Light^TM^ EdU Apollo567 In Vitro Kit (C10310-1, RiboBio, Guangzhou, P.R. China). Cells were treated as indicated for 24 h, incubated with EdU for 4 h, fixed in 4% paraformaldehyde for 30 min, permeabilized with 0.5% Triton X‑100, and processed according to the manufacturer’s instructions. Images were captured using an Olympus IX73 fluorescence microscope.

**Pyroptosis and apoptosis experiments**

To induce pyroptosis, Eca‑109 cells were pretreated with cycloheximide (CHX; 10 µg/mL; HY‑12320, MCE, NJ, USA) for 30 min, followed by stimulation with TNF‑α (20 ng/mL; 103‑01V, PrimeGene Bio‑Tech, MN, USA) for 24 h. To induce apoptosis, cells were treated with raptinal (20 µM; HY‑121320, MCE, NJ, USA) for 2 h. Pyroptosis and apoptosis were evaluated by immunoblotting for cleaved N‑terminal GSDMD and cleaved caspase‑3, respectively.

**Immunoprecipitation and co-immunoprecipitation**

TE-1 and Eca-109 cells were treated with 20 mM sodium racemic lactate or sodium L-lactate or sodium D-lactate for 24 h. Cells were lysed in NP-40 lysis buffer (P0013F, Beyotime, Shanghai, P. R. China) containing protease inhibitors (GRF102. Epizyme, Shanghai, P. R. China), for 15 min at 4℃. The soluble supernatant fractions were harvested by centrifugation at 12,000 × g for 10 min and then incubated with Pierce Protein G Magnetic Beads (88847, Thermo Fisher Scientific, California, USA) and anti-STAT3 antibodies (ab119352, Abcam, Cambridge, UK) overnight at 4℃. For co-immunoprecipitation experiments, HA-STAT3 and Flag-STAT3 plasmids were simultaneously transfected into cells using Lipofectamine 3000. Lysates were incubated with Flag beads (A36797, Thermo Fisher Scientific, California, USA) overnight at 4℃. Subsequently, beads were washed three times and proteins were eluted by heating for 10 min at 95℃ with 1× loading buffer prior to immunoblotting.

**Luciferase reporter assay**

Cells were seeded into 96-well plates at a density of 3×10^4^ per well and co-transfected with the pSTAT3-TA-luc (D2260-100 μg Beyotime, Shanghai, P. R. China) and pRL-TK (E2241, Promega, Wisconsin, USA) plasmid using Lipofectamine 3000. Cells were treated with 20 ng/mL IL-6 (10395-HNAE, SinoBiological, Beijing, P. R. China) and sodium racemic lactate (20-80 mM) for 24 h. Luciferase activity was measured using the Duo-Luciferase HS Assay Kit (LF004, GeneCopoeia, Maryland, USA) normalized to Renilla (pRL-TK).

**RT-qPCR**

Total RNA was extracted with an RNA simple Total RNA Kit (DP419, Tiangen, Beijing, P. R. China) and reverse transcribed using HiScript III All-in-one RT SuperMix (R333-01, Vazyme, Nanjing, P. R. China). qPCR was performed on a StepOnePlus system (Applied Biosystems, Foster City, California, USA) using SYBR Green (Q711-02, Vazyme, Nanjing, P. R. China). Relative expression was calculated by the 2^–ΔΔCт^ method withβ-actin as the internal control for mRNA assays. For bacterial quantification assays, 16S rRNA served as the internal reference where applicable. Primers were provided by Tsingke Biotechnology (Beijing, P. R. China) and Exon Biotechnology (Guangzhou, P. R. China).

**Western blotting**

Proteins were extracted using RIPA buffer (PC102, Beyotime, Shanghai, P. R. China) supplemented with protease inhibitors. A Nuclear and Cytoplasmic Protein Extraction Kit (P0027, Beyotime, Shanghai, P. R. China) was used to extract the nuclear and cytoplasmic proteins separately. Protein concentrations were determined via a BCA protein assay kit (ZJ102, Epizyme, Shanghai, P. R. China). Proteins were separated by SDS-PAGE, transferred to PVDF membranes (Bio-Rad, California, USA), incubated with primary antibodies at 4℃ overnight and secondary antibodies at room temperature for 1 h, and visualized using ECL (SQ201, Epizyme, Shanghai, P. R. China). β-actin (KC-5A08, KANGCHEN, Guangzhou, P.R. China) and histone H3 (4499S, CST, Massachusetts, USA) were used as controls for whole-cell lysates and nuclear proteins, respectively.

**Immunofluorescence**

Eca-109 cells were fixed in4% paraformaldehyde, permeabilized with 0.1% Triton X-100 for 30 min, and blocked with 1% BSA (9998S, CST, Massachusetts, USA) for 60 min. Cells were incubated with anti-STAT3 antibody overnight at 4℃, followed byAlexa Fluor^TM^ 594-conjugated goat anti-mouse IgG2a(γ2a) (A21135, Thermo Fisher Scientific, California, USA) at room temperature for 1 h and DAPI staining for 10 min. Images were acquired using an Olympus IX73 fluorescence microscope and a Nikon Eclipse Ti confocal microscope.

**MDA assay**

Intracellular MDA levels were measured using a Lipid Peroxidation MDA Assay Kit (S0131, Beyotime, Shanghai, P. R. China) according to the manufacturer’s instructions, with absorbance measured at 532 nm.

**Liperfluo staining**

Cells were treated as indicated for 24 h and stained with 10 μM Liperfluo (L248, DOJINDO, Kumamoto, Japan) in RPMI-1640 medium for 30 min at 37℃. Images were captured using an Olympus IX73 fluorescence microscope.

**BODIPY C11 assay**

Cells were treated as indicated for 24 h and stained with 5 μM C11-BODIPY 581/591 (D3861, Invitrogen, California, USA) for 30 min at 37℃ in the dark. Fluorescence shift (~590nm to ~510 nm) was analyzed on a BD FACSCelesta flow cytometer using the FITC channel.

**Lactate quantification**

L-lactate and D-lactate concentrations in tissues were measured using commercial assay kits (G02610, G02611, Westang Biotech, Shanghai, P. R. China) according to the manufacturer’s protocols. Absorbance was measured at 550 nm(L-lactate) and 490 nm (D-lactate). All the experiments were performed twice.

**Duolink^®^ Proximity ligation assay**

Tissue blocks of CATs and NATs were embedded in O.C.T. Compound (4583, Sakura, California, USA) and prepared as frozen tissue sections. The tissue slides were fixed in 4% paraformaldehyde and blocked with Duolink^®^ Blocking Solution at 37°C for 60 minutes. Primary antibody mixture (anti-STAT3 antibody and anti-lactyl-lysine rabbit mAb) was then added to each sample, and slides were incubated at 4℃ overnight. Subsequent steps followed the manufacturer’s instructions. Images were acquired using a Zeiss confocal microscope.

**Bacterial isolation and culture**

To isolate *Lactobacillus*, tumor tissues (approximately 0.5 g) were homogenized in1.5 mL sterile ice-cold PBS and digested with collagenase type 3 (A004180, Sangon, Shanghai, P. R. China) at 37℃ for 5 h with agitation. Digested material was filtered through a 70 μm nylon mesh. Tissue slurry (100 μL) was plated on MRS agar and incubated at 37℃under anaerobic or aerobic conditions for 3 or 5 days, respectively. *L. reuteri* was cultured as a positive control.

**Mouse xenograft experiments**

All mouse experiments were performed in accordance with the guidelines of the Ethical Committee of China Pharmaceutical University. The maximal tumor size allowed was 1500mm^3^. Animals were sacrificed when tumors reached this volume or earlier if predefined humane endpoints (more than 20% body weight loss) were observed. Male BALB/c nude mice (6-8 weeks) were purchased from GemPharmatech (Nanjing, P. R. China) and maintained under specific pathogen-free conditions with ad libitum access to food and water. Eca-109 cells (5×10^6^) were injected subcutaneously in 100 μL PBS. When tumors became palpable, mice were randomly allocated to four groups: (1) PBS (control); (2) *L. reuteri* in PBS; (3) Stattic; and (4) Stattic plus *L. reuteri*. Mice received *L. reuteri* (5×10^7^ CFU/mouse) via multipoint peritumoral injections twice weekly for 2 weeks. Stattic (25 mg/kg) was administered intraperitoneally once daily for 2 weeks. Control mice received matched volumes of PBS via peritumoral and intraperitoneal routes. Mice were euthanized at the end of treatment and tumors were harvested for downstream analyses. For experiments using the *ldhD*-deficient *L. reuteri* strain, mice received peritumoral injections of live mutant *L. reuteri* (5×10^7^ CFU) twice weekly. After one week, tumors from the wild-type and mutant groups were harvested for subsequent analyses. The tumor weights are presented as means ± SEM.

**Patient and public involvement**

Patients and/or the public were not involved in the design, conduct, reporting, or dissemination plans of this study.

**Supplementary Tables**

**Supplementary Table S1. Key resources used in the study**

**Supplementary Table S2. Demographic and clinical information of patients in the study**

**Supplementary Table S3. A list of detected contaminants in tumor microbiome 16S rRNA sequencing**

**Supplementary Table S4. Cox proportional-hazards models of overall survival and progression-free survival.**

**Supplementary Figures**

**Supplementary Figure S1. *Lactobacillus* is dominant in ESCC tumor tissues**

**
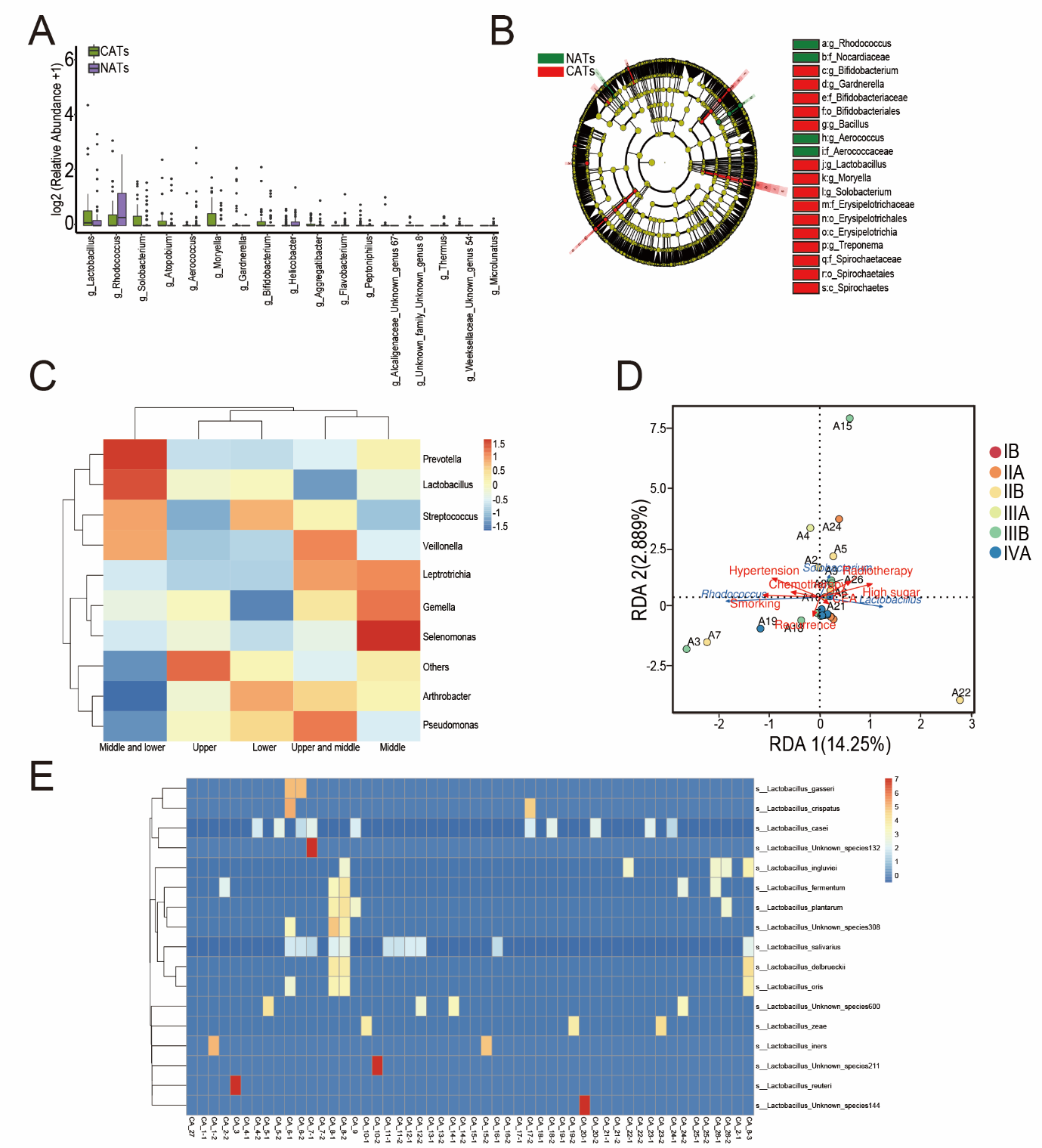
**

1. Differentially abundant taxa between CATs and NATs. Mann-Whitney U test (*P* < 0.05).
2. Cladogram showing taxa differentially enriched in CATs versus NATs.
3. Heatmap of the top 10 taxa across different anatomical locations of the esophagus.
4. Redundancy analysis of intratumor *Lactobacillus* abundance and relevant clinical variables.
5. Heatmap showing the relative abundance of detected *Lactobacillus* species across CAT samples.

Abbreviations: CATs, cancer tissues; NATs, normal adjacent tissues; PICRUSt, phylogenetic investigation of communities by reconstruction of unobserved states.

**Supplementary Figure S2. Intratumor heterogeneity of the ESCC-associated microbial community**

**
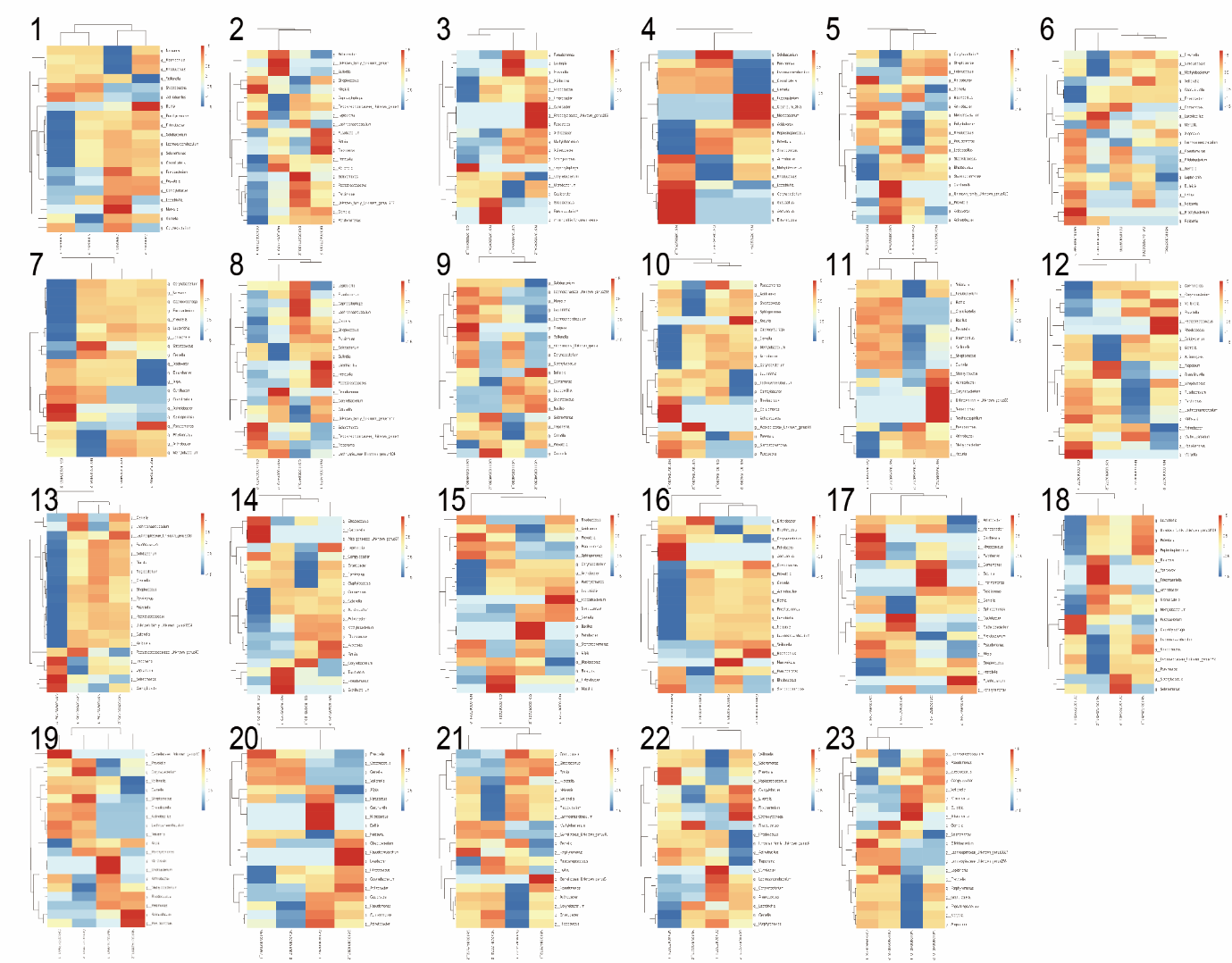
**

Microbiota profiles for each tumor biopsy and matched adjacent normal tissue from patient 1 to patient 23.

**Supplementary Figure S3. Related to Figure 3: Metabolic and phenotypical validation of *L. reuteri* -dependent ESCC stimulation**

**
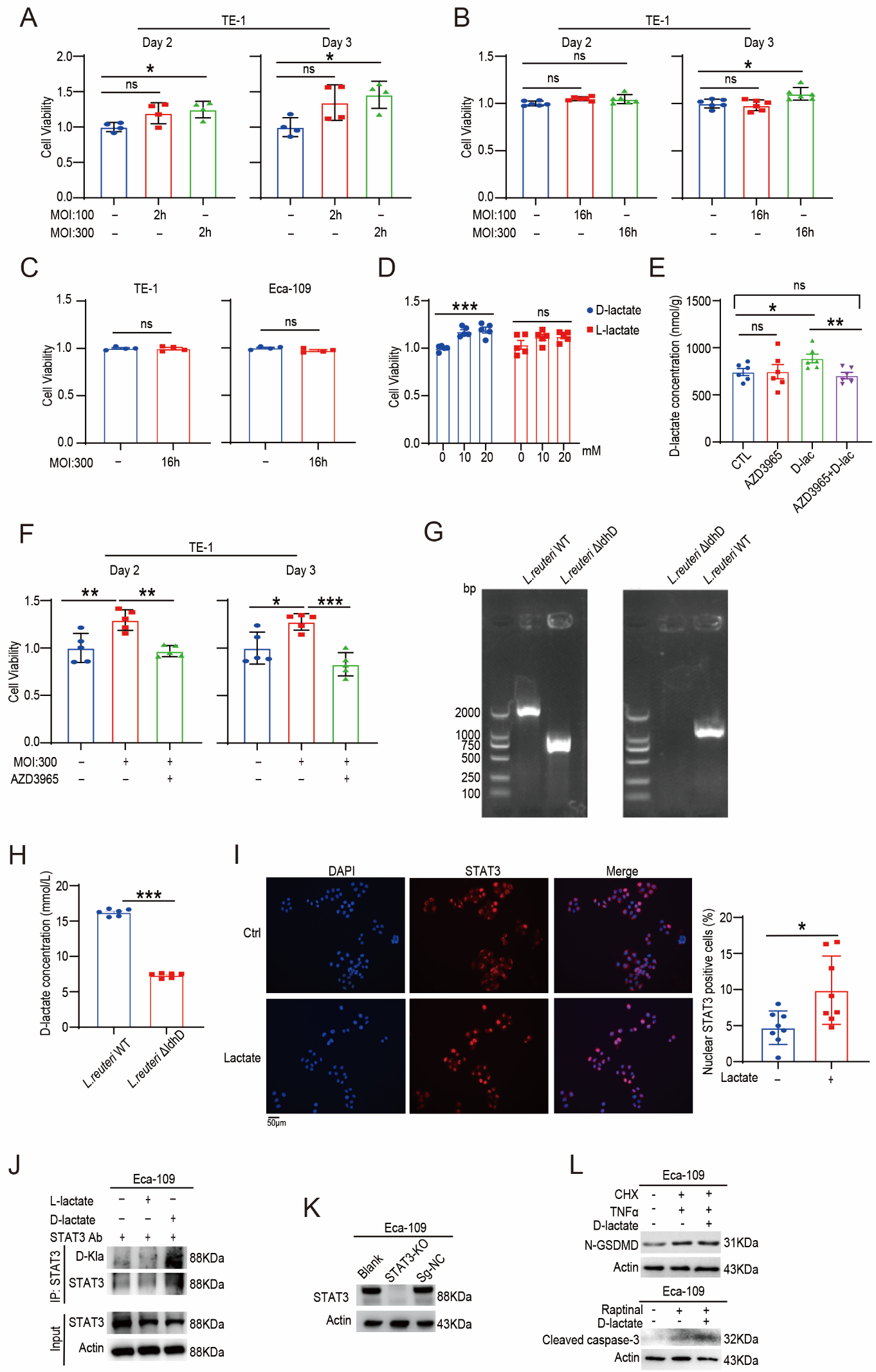
**

1. Viability of TE-1 cells treated with live *L. reuteri* at the indicated multiplicity of infection (MOI) for 2 h, assessed on day 2 and day 3 (*n* = 4).
2. Viability of TE-1 cells treated with live *L. reuteri* at the indicated MOI for 16 h, assessed on day 2 and day 3(*n* = 6).
3. Viability of TE-1 and Eca-109 cells treated with heat-killed *L. reuteri* at the indicated MOI for 16 h (*n* = 6).
4. Cell viability following exogenous treatment with varying concentrations (0, 10, 20 mM) of D-lactate or L-lactate for 24 h (n = 6).
5. Intracellular D-lactate concentrations in cells treated with the indicated treatments for 24 h (n=6).
6. Viability of TE-1 cells stimulated by *L. reuteri* supernatant, with or without AZD3965 co-treatment for 24 h, assessed on day 2 and day 3 (*n* = 5).
7. PCR verification of *ldhD* deletion in *L. reuteri*.
8. D-lactate concentrations in culture supernatant from wild-type and *ldhD*-deficient *L. reuteri* strains (n=6).
9. Representative immunofluorescence images and quantification of nuclear STAT3 localization in Eca-109 cells following lactate treatment. The bar graph indicates the percentage of cells with STAT3 nuclear translocation. Scale bar, 50 μm (*n* = 8).
10. Immunoprecipitation (IP) assay demonstrating stereospecific STAT3 modification. Treatment with exogenous D-lactate, but not L-lactate, specifically promoted STAT3 D-lactylation in Eca-109 cells.
11. Immunoblotting confirming STAT3 knockout in Eca-109 cells.
12. Immunoblotting showing pyroptosis- and apoptosis-associated cleavage markers in Eca‑109 cells following the indicated induction conditions.

Data are presented as means ± SEM. “n” represents the number of biologically independent replicates per group. Multi-group comparisons were evaluated globally utilizing one-way ANOVA coupled with Tukey’s post-hoc test. Statistical significance between two independent group was determined using an unpaired two-tailed Student’s t-test. * *P* < 0.05, ** *P* < 0.01, *** *P* < 0.001, ns, not significant.

Abbreviations: MOI: multiplicity of infection; WB, western blot; KO, knockout; Sg-NC, short guide RNA negative control; D-Kla, D-lactyllysine.

**Supplementary Figure S4. STAT3 lactylation regulates GPX4/FTH1 expression and links D-lactate levels to ESCC prognosis
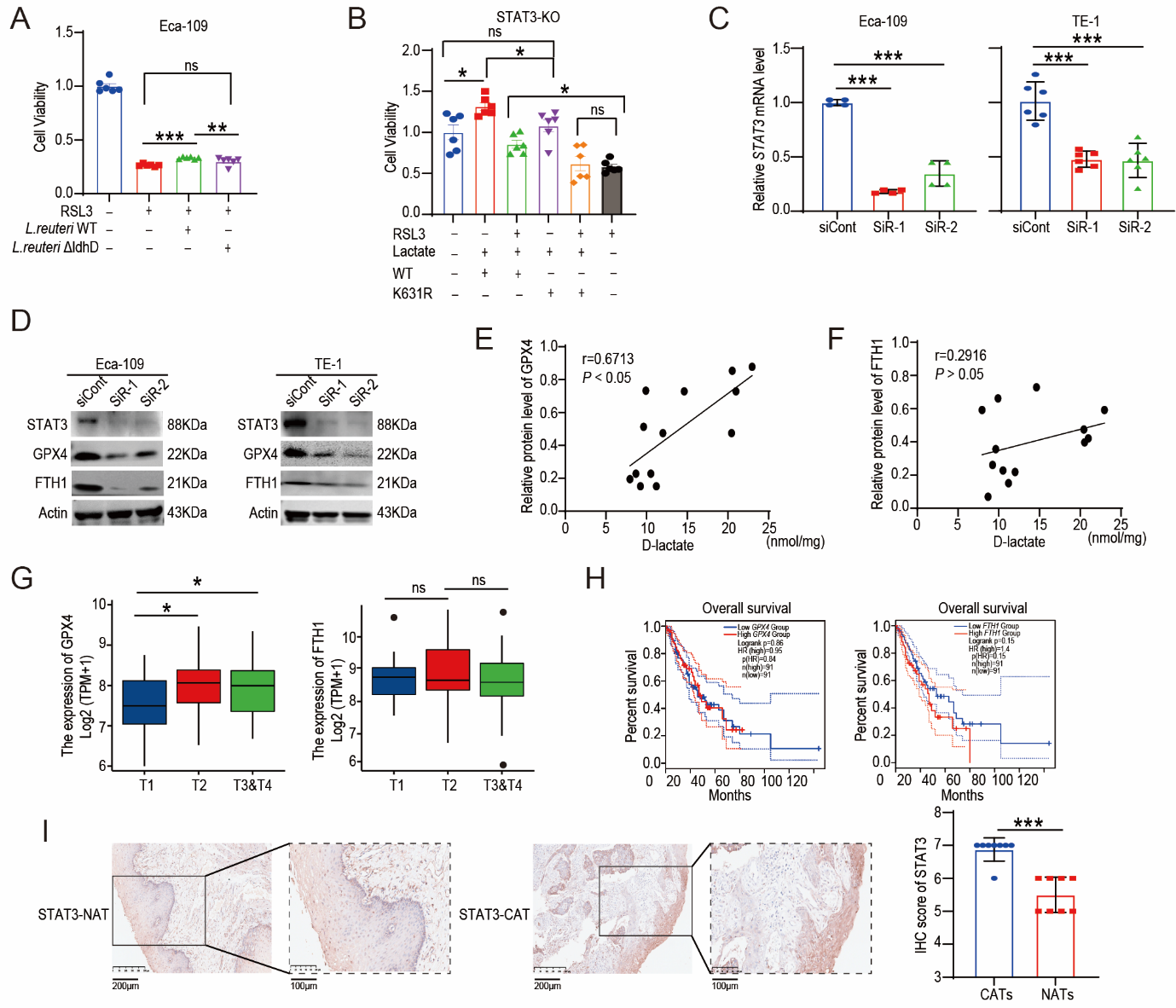
**

(A) Viability of Eca-109 cells treated with RSL3 (100 nM, 24 h) combined with the wild-type or *ldhD*-deficient *L. reuteri* strain for 16 h (*n* = 6).

(B) Viability of STAT3-KO Eca-109 cells reconstituted with wild-type STAT3 or STAT3 K631R plasmid and treated with RSL3 (100 nM) and/or 20 mM lactate for 24 h (*n* = 6-8).

(C) RT-qPCR validation of *STAT3* knockdown in Eca-109 and TE-1 cells transfected with control or STAT3 siRNAs (*n* = 4 or 6).

(D) Immunoblotting of the indicated proteins in Eca-109 and TE-1 cells transfected with control or STAT3 siRNAs.

(E, F) Spearman’s rank correlation analyses exploring the quantitative relationship between intratumor D-lactate concentrations and relative protein levels of GPX4 (E) and FTH1 (F) in CATs.

(G) *GPX4* and *FTH1* expression across pathologic stages in ESCC using TCGA database.

(H) Kaplan-Meier survival analysis of ESCC patients stratified by high versus low GPX4 or FTH1 expression using GEPIA2 (cut-off are indicated in the plot). Statistical significance between curves was evaluated utilizing the log-rank test.

(I) IHC staining of STAT3 in CATs and NATs, with semi-quantitative IHC scores shown on the right. Scale bars, 200 μm and 100 μm from left to right, respectively (*n* = 8).

Data are presented as means ± SEM. “n” represents the number of independent replicates. Multi-group comparisons were evaluated globally utilizing One-way ANOVA coupled with Tukey’s post-hoc test. Statistical significance between two independent groups was determined using an unpaired two-tailed Student’s t-test, whereas paired clinical samples were analyzed using a paired two-tailed Student’s t-test (I). * *P* < 0.05, *** *P* < 0.001, ns, not significant.

Abbreviations: CATs, cancer tissues; NATs, normal adjacent tissues; RT-qPCR, real-time quantitative PCR; ESCC, esophageal squamous cell carcinoma; IHC, immunohistochemistry.

**Supplementary Figure S5. *GPX4* and *FTH1* expression patterns across multiple tumor types**

**
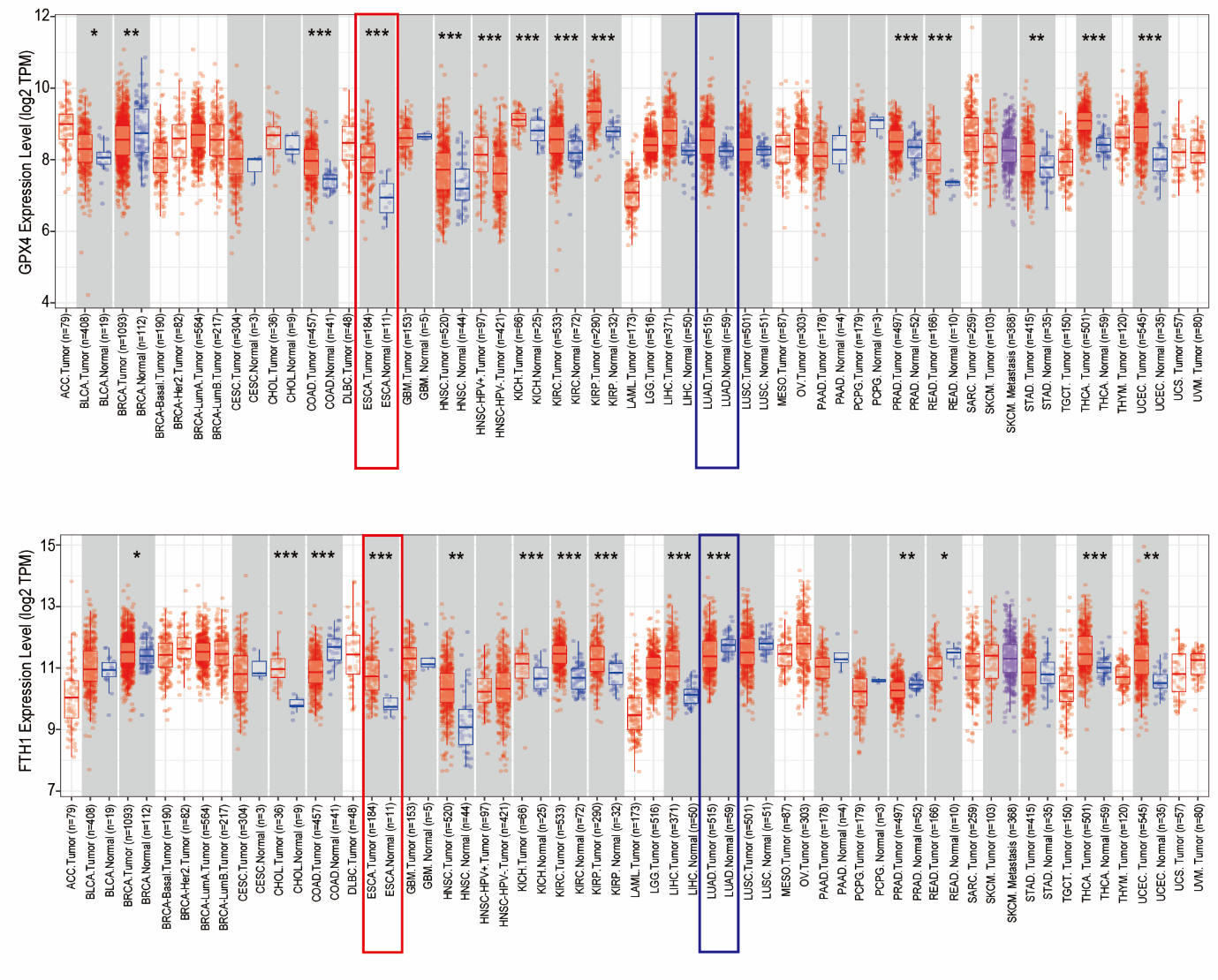
**

Expression profiles of *GPX4* and *FTH1* across multiple tumor types were obtained from the TIMER2.0 resource (http://timer.comp-genomics.org/).*** *P* < 0.001.

Abbreviations: ESCA, esophageal squamous cell carcinoma; LUAD, lung adenocarcinoma.

**Supplementary Figure S6.** **Distinct *Lactobacillus* patterns in LUAD and D-lactate specificity in ESCC
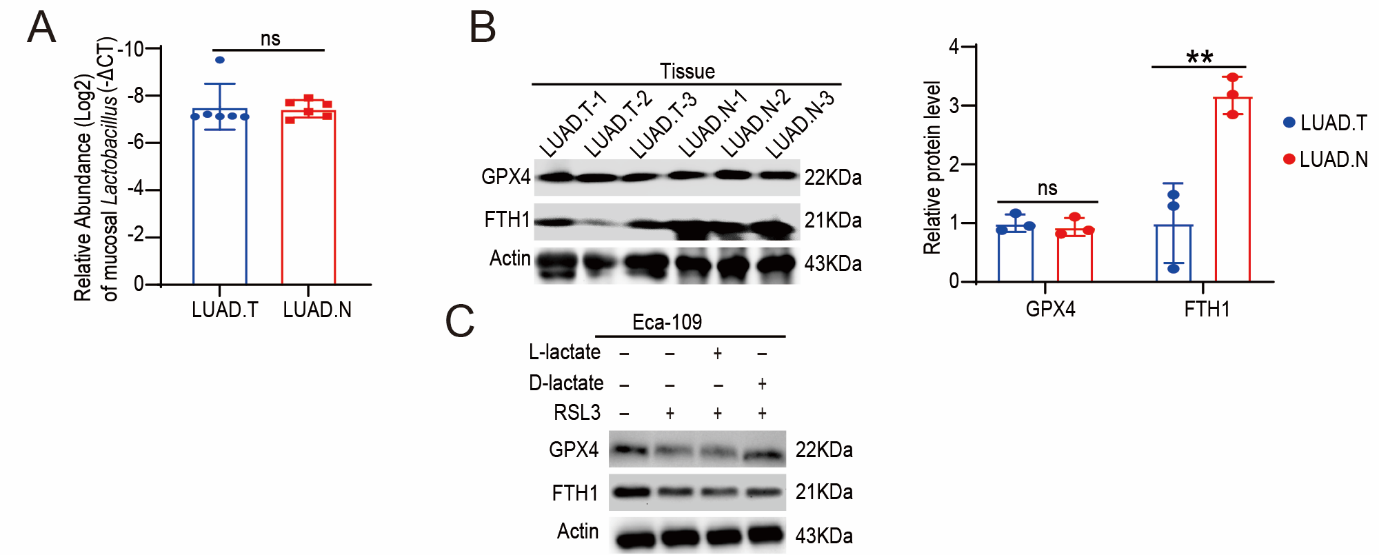
**

(A) qPCR quantification of *Lactobacillus* abundance in LUAD tumor tissues (LUAD.T) and paired adjacent normal tissues (LUAD.N). Mann-Whitney U test (*n* = 3 biological pairs, technical duplicates).

(B) Immunoblotting of GPX4 and FTH1 in LUAD.T and LUAD.N tissues, with density quantification shown. Paired two-tailed Student’s t-test (*n* = 3).

(C) Immunoblotting evaluating the differential potencies of enantiomeric L-lactate (20 mM) versus bacteria-derived D-lactate (20 mM) in mitigating RSL3-induced GPX4 and FTH1 protein degradation in Eca-109 cells.

Data are presented as means ± SEM. “n” represents the number of tissue samples or protein

bands in each group. ** *P* < 0.01, ns, not significant.

Abbreviations: ESCC, esophageal squamous cell carcinoma; qPCR, quantitative PCR; LUAD, lung adenocarcinoma.

**Supplementary Figure S7. Illustration of the role of *Lactobacillus* in ESCC development**

**
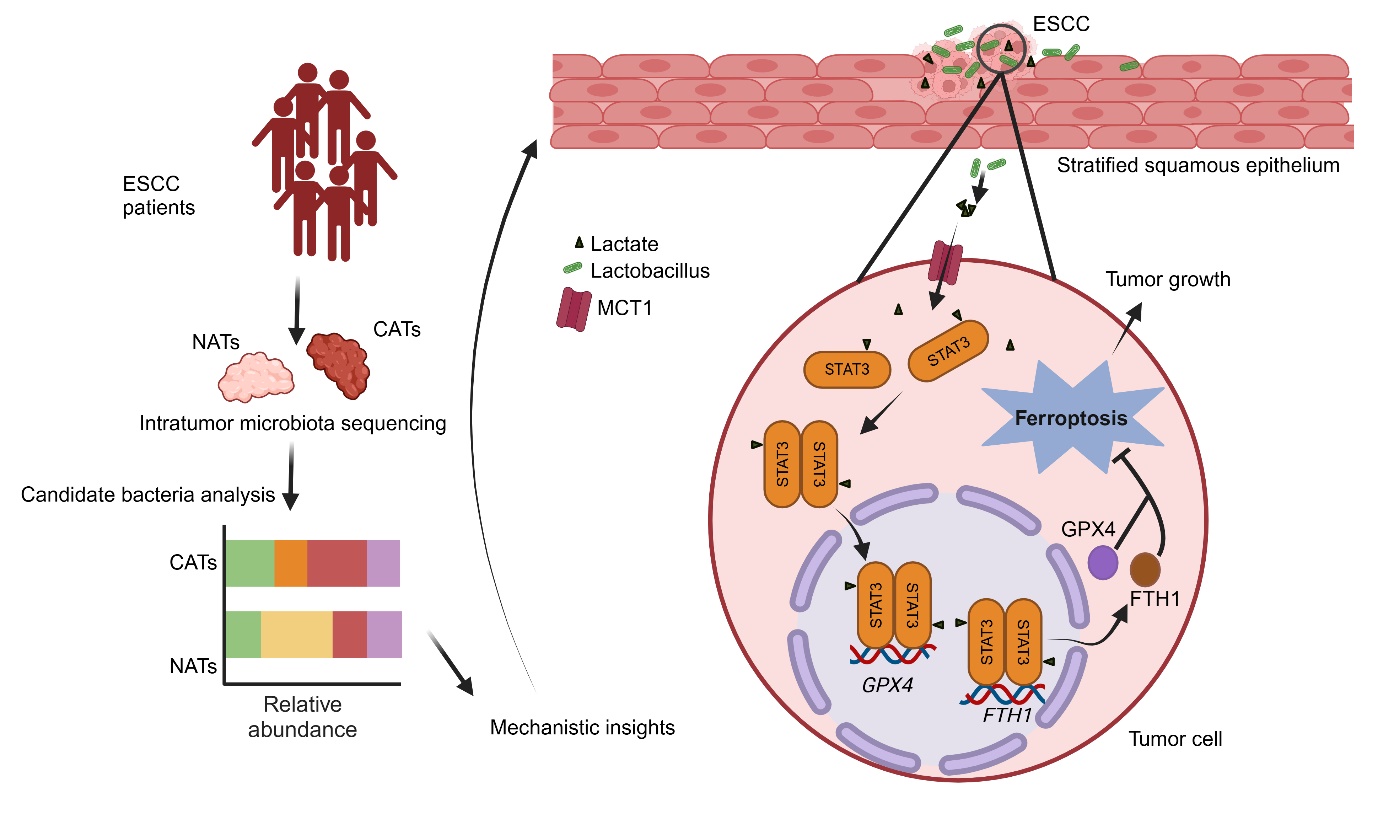
**

Multi-regional profiling of tissue-resident microbiota in ESCC identified increased *Lactobacillus* abundance in CATs compared with NATs. Tumor-resident *Lactobacillus* (including *L. reuteri*) elevates lactate levels within the tumor microenvironment. Lactate enters tumor cells via monocarboxylate transporter 1 (MCT1) and induces STAT3 lactylation at K631, thereby enhancing STAT3 dimerization and nuclear translocation to upregulate ferroptosis suppressors glutathione peroxidase 4 (GPX4) and ferritin heavy chain 1 (FTH1). This axis suppresses ferroptosis and promotes ESCC tumor growth. The schematic was created with BioRender.

**References**

1. Nejman D, Livyatan I, Fuks G, Gavert N, Zwang Y, Geller LT*, et al.* The human tumor microbiome is composed of tumor type-specific intracellular bacteria. Science 2020;**368**:973-80.

2. Hong J, Guo F, Lu SY, Shen C, Ma D, Zhang X*, et al.* F. nucleatum targets lncRNA ENO1-IT1 to promote glycolysis and oncogenesis in colorectal cancer. Gut 2021;**70**:2123-37.

3. Fu A, Yao B, Dong T, Chen Y, Yao J, Liu Y*, et al.* Tumor-resident intracellular microbiota promotes metastatic colonization in breast cancer. Cell 2022;**185**:1356-72 e26.

4. Ouyang S, Li H, Lou L, Huang Q, Zhang Z, Mo J*, et al.* Inhibition of STAT3-ferroptosis negative regulatory axis suppresses tumor growth and alleviates chemoresistance in gastric cancer. Redox Biol 2022;**52**:102317.

5. Zhou CB, Pan SY, Jin P, Deng JW, Xue JH, Ma XY*, et al.* Fecal Signatures of Streptococcus anginosus and Streptococcus constellatus for Noninvasive Screening and Early Warning of Gastric Cancer. Gastroenterology 2022;**162**:1933-47e18.

6. Jiang SS, Xie YL, Xiao XY, Kang ZR, Lin XL, Zhang L*, et al.* Fusobacterium nucleatum-derived succinic acid induces tumor resistance to immunotherapy in colorectal cancer. Cell Host Microbe 2023;**31**:781-797.e9.
